# Transcriptomic and metabolic analyses revealed the modulatory effect of vernalization on glucosinolate metabolism in radish (*Raphanus sativus* L.)

**DOI:** 10.1038/s41598-021-03557-5

**Published:** 2021-12-15

**Authors:** Adji Baskoro Dwi Nugroho, Sang Woo Lee, Aditya Nurmalita Pervitasari, Heewon Moon, Dasom Choi, Jongkee Kim, Dong-Hwan Kim

**Affiliations:** 1 Department of Plant Science and Technology, Chung-Ang University, Anseong, 17546 Republic of Korea; 2 Research Center for Plant Plasticity, Seoul National University, Seoul, 08826 Republic of Korea

**Keywords:** Molecular biology, Plant sciences

## Abstract

Vernalization is the process by which long-term cold like winter triggers transition to flowering in plants. Many biennial and perennial plants including *Brassicaceae* family plants require vernalization for floral transition. Not only floral transition, but dynamic physiological and metabolic changes might also take place during vernalization. However, vernalization-mediated metabolic change is merely investigated so far. One of secondary metabolites found in *Brassiceceae* family plants is glucosinolates (GSLs). GSLs provides defense against pathogens and herbivores attack in plants and also exhibits inhibitory activity against human cancer cell. Profiles of GSLs are highly modulated by different environmental stresses in *Brassciaceae* family plants. To grasp the effect of vernalization on GSLs metabolic dynamics in radish (*Raphanus sativus* L.), we performed transcriptomic and metabolic analysis during vernalization in radish. Through transcriptome analysis, we found many GSLs metabolic genes were significantly down-regulated by vernalization in radish plants. Ultra-High Performance Liquid Chromatography analysis also revealed that GSLs compounds were substantially reduced in vernalized radish samples compared to non-vernalized radish samples. Furthermore, we found that repressive histone modification (i.e. H3K27me3) is involved in the modulation of GSLs metabolism via epigenetic suppression of *Glucoraphasatin Synthase 1* (*GRS1*) during vernalization in radish. This study revealed that GSLs metabolism is modulated by vernalization, suggestive of a newly identified target of vernalization in radish.

## Introduction

Plants, as sessile organisms, adapt to constantly changing environmental conditions by adjusting their growth and developmental programs^1^. Under biotic (herbivore and pathogen) and abiotic (salt, heat, and wounding) stress conditions, plants cope by regulating several metabolic processes and pathways^2^. For instance, plants synthesize several defensive compounds such as alkaloids, benzoxanoids, terpenoids, and glucosinolates (GSLs)^3^ to cope with stress and harsh environmental conditions. Among these secondary metabolites, GSLs and their hydrolyzed products, which are mainly found in plants of family Brassicaceae, including radish, play a defensive role against diverse stresses. Additionally, GSLs have attracted considerable research attention because of their anticarcinogenic activity against human cancer cells^4–6^. Based on their amino acid precursors, GSLs can be classified into three groups: aliphatic, indolic, and aromatic GSLs. Aliphatic GSLs are derived from alanine, leucine, isoleucine, valine, and methionine, whereas indolic and aromatic GSLs are derived from tryptophan and phenylalanine or tyrosine, respectively^7^.

**Figure 1:**
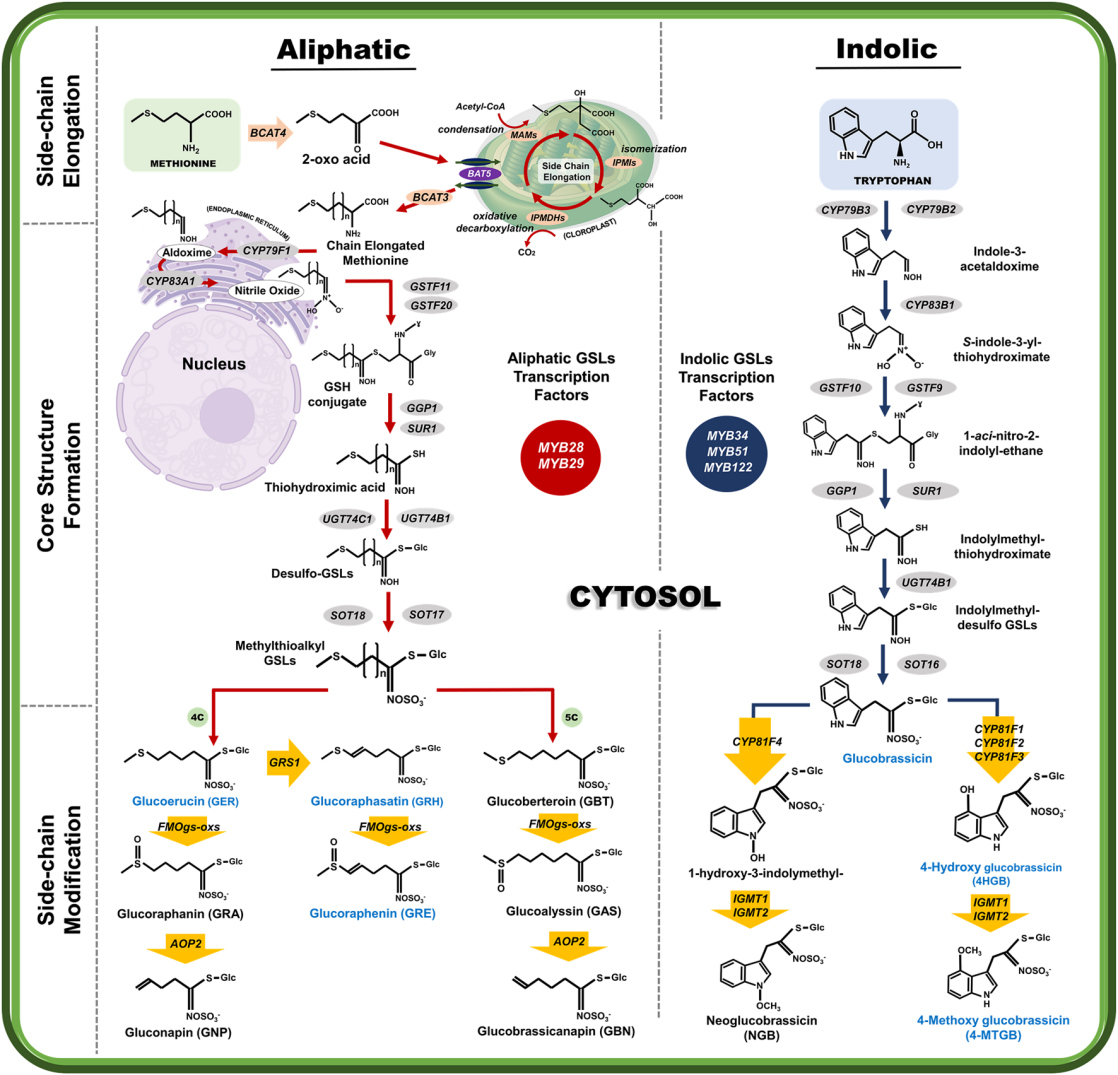
Schematic pathways of aliphatic and indole GSLs biosynthesis in plants of family *Brassica* as well as in *R. sativus*. Green and blue arrows represent pathways for aliphatic and indolic GSLs biosynthesis, respectively. GSLs detected in this study are presented with blue color, whereas undetected GSLs are indicated with black color.

Among plants of family *Brassicaceae*, radish (*Raphanus sativus* L.) is particularly important because of its edible tuberous root vegetable, which is consumed globally. Additionally, radish is a rich source of aliphatic GSLs^8^, particularly glucoraphasatin (GRH), which accounts for approximately 60–90% of aliphatic GSLs in radish^9,10^. Therefore, studies have examined the biosynthesis of GRH in radish. Recent findings have shown that GRH biosynthesis mainly occurs in the leaf tissue of radish, but are translocated to the root^11,12^. Glucoraphasatin synthase 1 (*RsGRS1*) gene, which encodes 2-oxoglutarate-dependent dioxygenase, regulates the conversion of glucoerucin (GER) to GRH through dehydrogenation activity (Fig. 1)^12^. The transcript level of *GRS1* was highly detected in the leaf tissue compared to the other tissues of radish^11,12^. The expression of *RsGRS1* increases during the vegetative stage in radish, but decreases after the plant has attained the reproductive stage, as evidenced by the lower concentration of GRH in reproductive tissues such as flower and pod compared with that of vegetative tissues. Further conversion of GRH to glucoraphenin (GRE) is catalyzed by a subgroup of flavin-containing monooxygenase family proteins referred to as FMOgs-OXs (Fig. 1).

Under prolonged cold conditions, such as winter, there is an accelerated developmental shift from vegetative stage to reproductive stage in biennial and perennial plants, and this phenomenon is termed vernalization. Many Brassicaceae family plants, including cabbage, broccoli, rapeseed, and radish require vernalization for floral transition^13,14^. Vernalization not only improves flowering but also cause physiological and metabolic changes in plants. In a previous study, vernalization modulated the expression of many metabolic genes in *Arabidopsis* model plant^15^. Since it is already established that environmental stimuli such as drought and high temperatures can influence the production of GSLs metabolites^16^, vernalization also might affect the biosynthesis of GSLs metabolism in many Brassicaceae family plants. However, studies are yet to examine the effect of vernalization in the biosynthesis of GSLs in radish. Therefore, the aim of this study was to examine the effect of vernalization on the biosynthesis of aliphatic and indolic GSLs in radish. The aliphatic and indolic GSLs contents of the leaves and roots of radish were examined by high-performance liquid chromatography (HPLC). Additionally, the genetic mechanism underlying GSLs biosynthesis was examined using RNA sequencing technology.

## Results

### GSLs profiles of non-vernalized and vernalized radish

**Figure 2:**
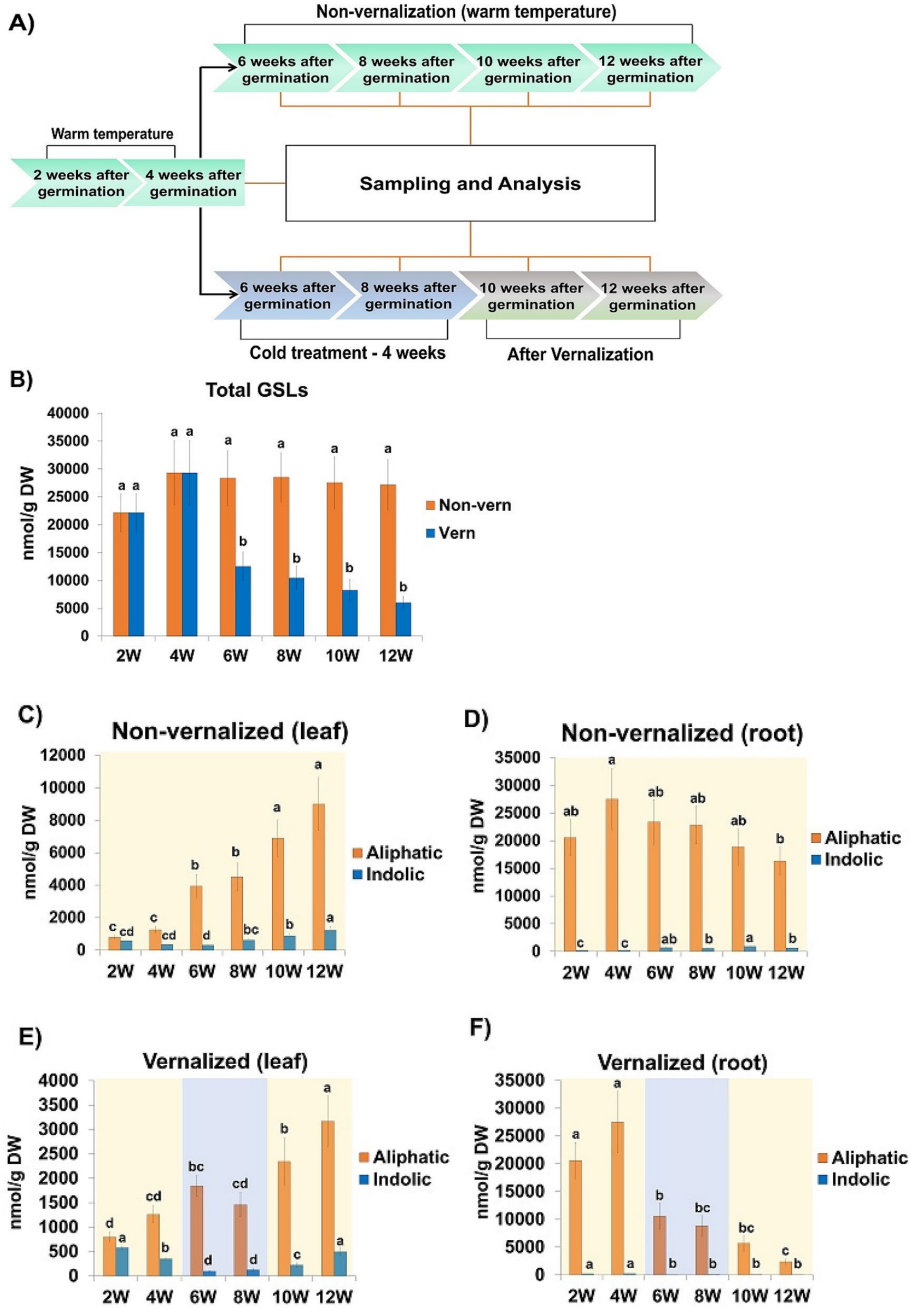
Aliphatic and indolic GSLs content of vernalized and non-vernalized *R. sativus* during vegetative growth. (**A**) Schematics of the study process. For vernalization treatment, 4-week-old plants were transferred to cold room and grown for 4 weeks (6- and 8-week stages) and transferred back to the ambient temperature (22 °C) for additional 4 weeks (10- and 12-week stages). In contrast. non-vernalized plants were grown under ambient temperature conditions throughout the experimental period. (**B**) Total GSLs content of non-vernalized (orange) and vernalized (blue) plant along different time points (**C**) Total aliphatic and indolic GSLs content of the leaves of non-vernalized plants. (**D**) Total aliphatic and indolic GSLs content of the roots of non-vernalized plants. (**E**) Total aliphatic and indolic GSLs content of the leaves of vernalized plants. (**F**) Total aliphatic and indolic GSLs content of the roots of vernalized plants. Data were presented as mean ± standard deviation (SD) (n = 3). Statistically significant differences were determined by one-way ANOVA and Tukey’s post hoc test (*p* < 0.05).

In the present study, the GSLs content and profiles of vernalized and non-vernalized radish were examined (Fig. 2A). Overall, the seedlings were sampled for analysis at 2 and 4 weeks of age (before vernalization treatments), 6 and 8 weeks of age (during vernalization treatment), and 10 and 12 weeks of age (after vernalization treatment). For the vernalization treatment group, 4-week-old seedlings were exposed to cold (4 °C) for 4 weeks and then returned to warm temperature (22 °C) for 4 weeks (Fig. 2A). Seedlings in the non-vernalized control group were not exposed to cold temperature. GSLs profile analysis of vernalized and non-vernalized seedlings by U-HPLC identified seven GSLs, among which four were aliphatic (GER, PGT, GRE, and GRH), while three were indolic (4-HGB, GBS, and 4-MTGB) (Supplementary Fig. S2).

### Total GSLs content of the leaf and root along different time points

Furthermore, the total GSLs (aliphatic + indolic GSLs) content of non-vernalized and vernalized plants was examined at six time points (Fig. 2B). The GSLs content of non-vernalized seedlings was not significantly different at the time points examined (2, 4, 6, 8, 10, and 12 weeks). In contrast, vernalization significantly decreased the GSLs content of seedlings in the vernalization treatment group. Additionally, the GSLs content of the roots and leaves of vernalized and non-vernalized seedlings was examined. Results showed that the total GSLs content (aliphatic + indolic GSLs) of the leaves of non-vernalized seedlings exhibited an increasing trend with plant age (2–12 week) (Supplementary Fig. S2A). However, although the GSLs content of the leaves of seedlings in the vernalization treatment group significantly increased after vernalization (6 weeks), the increase was not as evident as that of the leaves of non-vernalized plants (Supplementary Fig. S2A). Regarding the root tissue, there was no significant difference in the GSLs content of the roots of non-vernalized plants along the time points (Supplementary Fig. S2B). In contrast, vernalization significantly reduced the root GSLs content of the plants. These results indicated that vernalization reduced GSLs biosynthesis in radish.

### Aliphatic and indolic GSLs profiles of radish leaf and root along time points

In the present study, the aliphatic and indolic GSLs contents of the leaves and roots of vernalized and non-vernalized plants were examined. The aliphatic GSLs content of both the leaves and roots of vernalized and non-vernalized plants was considerably higher than the indolic GSLs content (Fig. 2C–F). At 2 weeks old, the aliphatic GSLs content of the root (198.60 nmol/g) of non-vernalized plants was approximately 103.5-fold higher than the indolic GSLs content (20,563.28 nmol/g) (Fig. 2D). Additionally, the aliphatic GSLs content of the roots was significantly higher than that of the leaves in both non-vernalized and vernalized plants. Overall, these results indicated that the GSLs content of the roots of radish was higher than that of the leaves, and that aliphatic GSLs constituted the highest proportion of GSLs in both the leaves and roots of radish.

### Vernalization decreases the total GSLs content of radish

Compared with non-vernalized plants, there was a significant decrease in the total GSLs content of the leaves and roots of vernalized plants (Fig. 2B). Although vernalization decreased the total GSLs content of the roots and leaves, it had varying effects on the aliphatic and indolic GSLs content of the leaves and roots. The aliphatic GSLs content of the leaves of vernalized plants significantly increased along the time points, whereas the indolic acid content exhibited a quadratic pattern, with the lowest levels obtained at 6 and 8 weeks of age, then increasing afterwards (Fig. 2E). In contrast, the aliphatic GSLs content of the roots of vernalized plants decreased significantly along the time points, with the lowest value obtained at 12 weeks of age (Fig. 2F).

### Vernalization decreases the glucoraphasatin (GRH) content of radish

**Figure 3:**
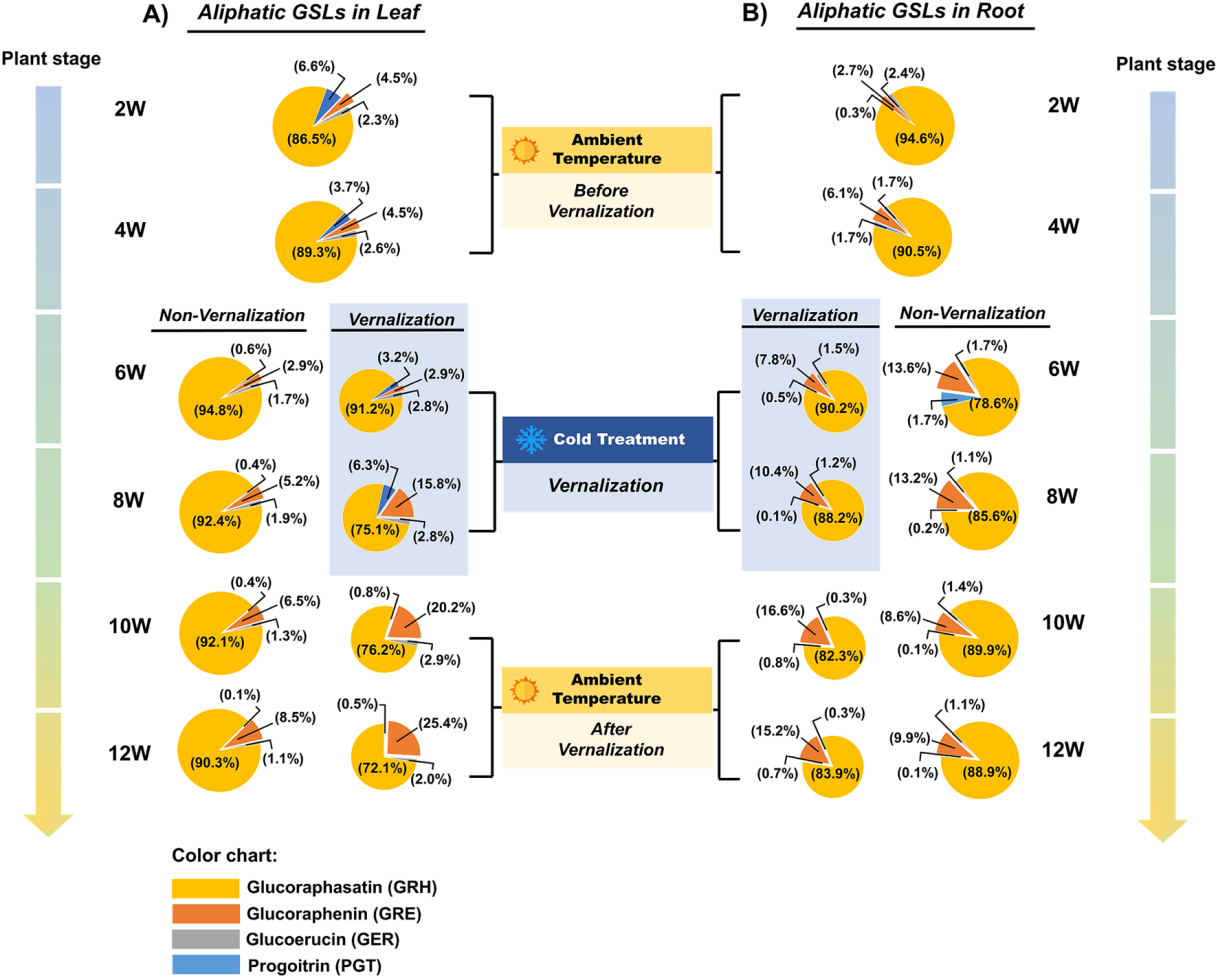
The aliphatic GSLs content and profiles of the leaves (**A**) and roots (**B**) of vernalized and non-vernalized *R. sativus*. The smaller size of pie-graph for the vernalization group indicated lower total aliphatic GSLs content compared with the non-vernalization group. Data were presented as mean ± standard deviation (SD) (n = 3). Statistically significant differences were determined by one-way ANOVA and Tukey’s post hoc test (*p* < 0.05). *PGT* Progoitrin, *GRE* Glucoraphenin, *GER* Glucoerucin, *GRH* Glucoraphasatin.

Since aliphatic GSLs accounted for a major proportion of GSLs in radish, we examined the aliphatic GSLs profile of the seedling. Among four aliphatic GSLs compounds detected, glucoraphasatin (GRH) constituted the largest proportion of aliphatic GSLs. GRH accounted for approximately 72–94% of aliphatic GSLs in the roots and leaves of both vernalized and non-vernalized plants. Although GRH constituted a large proportion of aliphatic GSLs in the leaves and roots of both vernalized and non-vernalized plants at the time points examined, there was a decrease in their proportion at 8, 10, and 12 weeks (Fig. 3A,B). Overall, the GRH content of the leaves of non-vernalized plants was higher than that of vernalized plants at all time points. In contrast, vernalization increased the GRE content of the leaves, but decreased the GRE content of the roots of the seedlings. Additionally, the GRE content of the roots of non-vernalized seedlings was higher than that of vernalized seedling at 10 and 12 weeks. These results indicated that although vernalization reduced the total aliphatic GSLs content of radish, it increased the GRE content.

### Indolic GSLs content of non-vernalized and vernalized plants

The indolic GSLs content of the leaves of non-vernalized and vernalized plants exhibited a quadratic pattern along the time points, with the lowest values obtained at 6 weeks, and increasing afterwards. However, the indolic GSLs content of the leaves of non-vernalized plants was higher than that of vernalized plants at all time points (Supplementary Fig. S3A and S3B), indicating that vernalization decreased the indolic GSLs content of the leaves. Additionally, the indolic GSLs content of the roots of non-vernalized plants increased along the time points, whereas that of the roots of vernalized plant decreased along the time points. The reduction of indolic GSLs content by vernalization was more evident in the roots. These results indicated that the indolic GSLs content of radish increased with age.

### Indolic GSLs content of the leaf and root of radish

**Figure 4:**
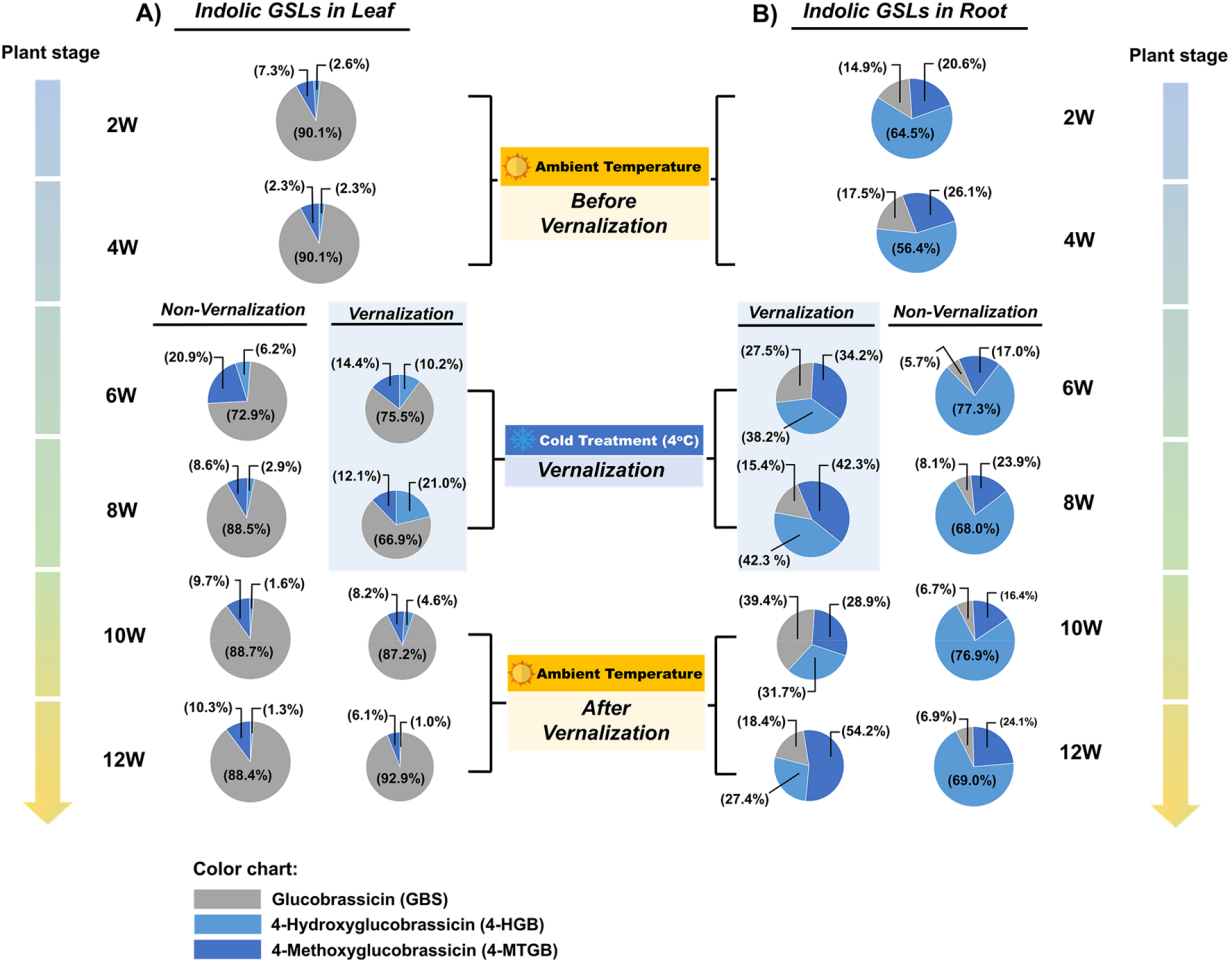
The indolic GSLs content and profiles of the leaves (**A**) and roots (**B**) of vernalized and non-vernalized *R. sativus*. The smaller size of pie-graph for the vernalization group indicated lower total indolic GSLs content compared with the non-vernalization group. Data were presented as mean ± standard deviation (SD) (n = 3). Statistically significant differences were determined by one-way ANOVA and Tukey’s post hoc test (*p* < 0.05). *GBS* Glucobrassicin, *4-HGB* 4-Hydroxyglucobrassicin, *4-MTGB* 4-Methoxyglucobrassicin.

The indolic GSLs profiles of the roots and leaves of vernalized and non-vernalized seedlings were examined. There were significant differences in the indolic GSLs profiles of the leaves and roots of vernalized and non-vernalized plants. GBS constituted the largest proportion (72.9–92.9%) of indolic GSLs in the leaves of both vernalized and non-vernalized seedlings, followed by 4-MTGB and 4-HGB (Fig. 4A). In contrast, the indolic GSLs profile of the roots of vernalized seedlings was dynamic, with vary proportions of the three GSLs at different time points. However, 4-HGB constituted the largest proportion of indolic GSLs in the roots of non-vernalized seedlings (Fig. 4B).

### Illumina sequencing and mapping to radish reference genome

Furthermore, the transcriptomes of the leaves and roots of radish were examined at three time points: BV (before-vernalized), V (during vernalized), and AV (after-vernalized). Three biological replicates were used for each time point, making a total of nine RNA-sequencing libraries. Total reads and mapped reads on the radish reference genome (http://www.nodai-genome-d.org/) are shown in Supplementary Table S3. Multi-dimensional scaling (MDS) plot of the nine RNA-seq reads showed that samples of the same treatment clustered together, validating the RNA-seq data (Supplementary Fig. S4A).

### Identification of differentially expressed genes (DEGs)

**Figure 5:**
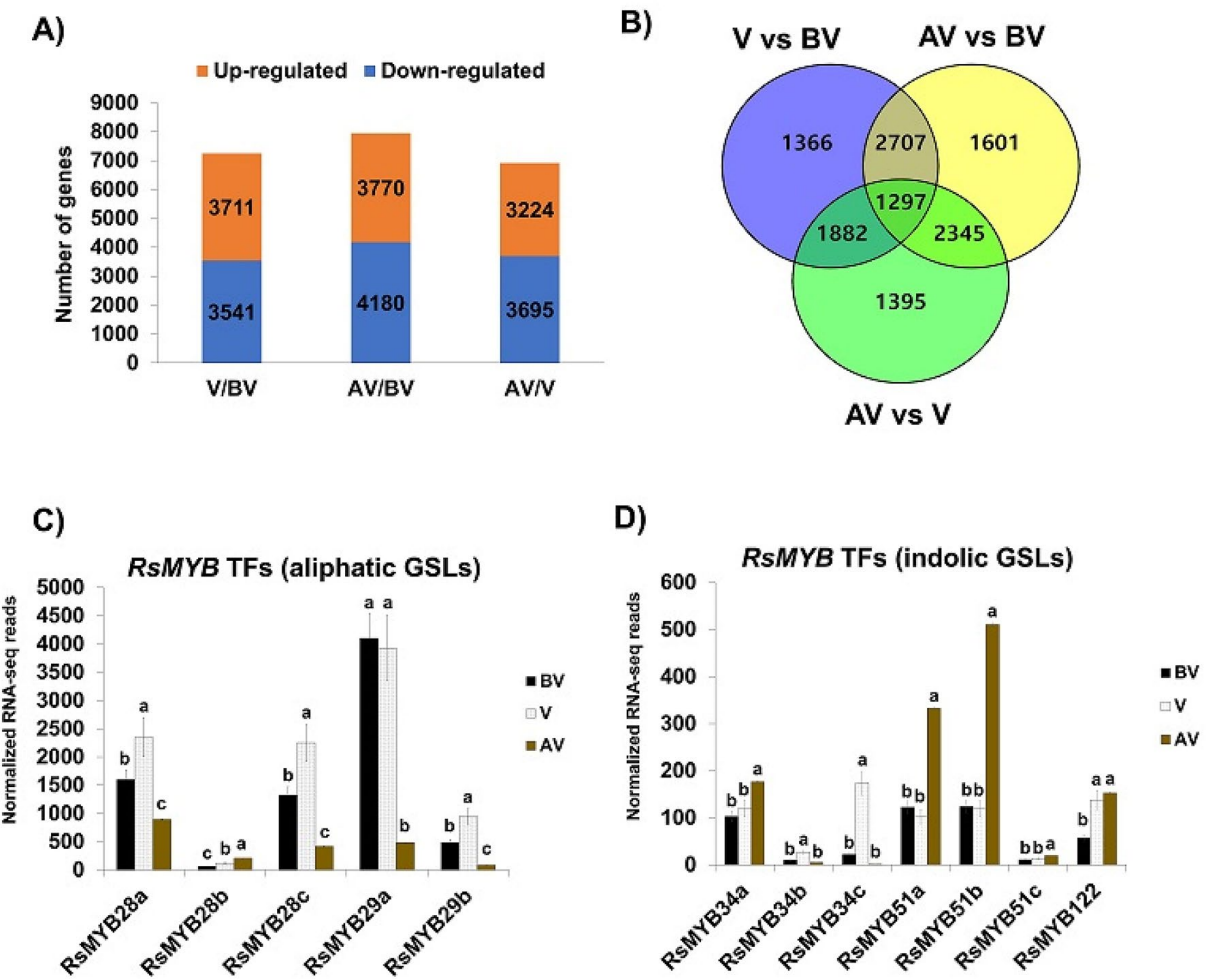
Identification of differentially expressed genes (DEGs) among the three different vernalization time point. (**A**) The number of upregulated and downregulated genes in response to vernalization. The number of genes in each time point was compared to each other (*BV* before-vernalized, *V* vernalized, *AV* after vernalized). (**B**) Venn-diagram of DEGs between different vernalization time point. A total of 2,707 DEGs were common to V and AV compared with NV. A total of 1,601 DEGs were common to AV compared with AV vs BV. Venn diagram data was generated using VENNY webtool (version 2.1.0) (https://bioinfogp.cnb.csic.es/tools/venny/). ^53^. (**C**,**D**). Normalized RNA-seq reads counts of *RsMYB* TFs genes involved in aliphatic and indolic GSLs biosynthesis along three time points (*BV* before-vernalized, *V* vernalized, *AV* after-vernalized).

In the present study, we compared the number of DEGs between each time points: V/BV, AV/BV, and AV/V. As shown in Fig. 5A, 3,711 upregulated and 3,541 downregulated genes were identified between V/BV (Supplementary Fig. S4B), whereas 3,770 upregulated and 4180 downregulated genes were identified between AV/BV. Additionally, 3224 upregulated and 3695 downregulated genes were identified between AV/V. These results indicated that vernalization significantly influenced the transcriptome of the leaves and roots of radish plant, which was similar to findings in *Arabidopsis*^15^. Furthermore, the number of overlapping DEGs between the time points was illustrated using a Venn diagram (Fig. 5B)*.* A total of 2,707 DEGs were common to V and AV, whereas 1,601 DEGs were expressed in only AV. Overall, 1297 DEGs were common to all the comparison groups. These results indicated that vernalization significantly affected the RNA profiles of the seedlings.

### Transcriptional profile of *MYB* transcription factor in GSLs biosynthesis

**Figure 6:**
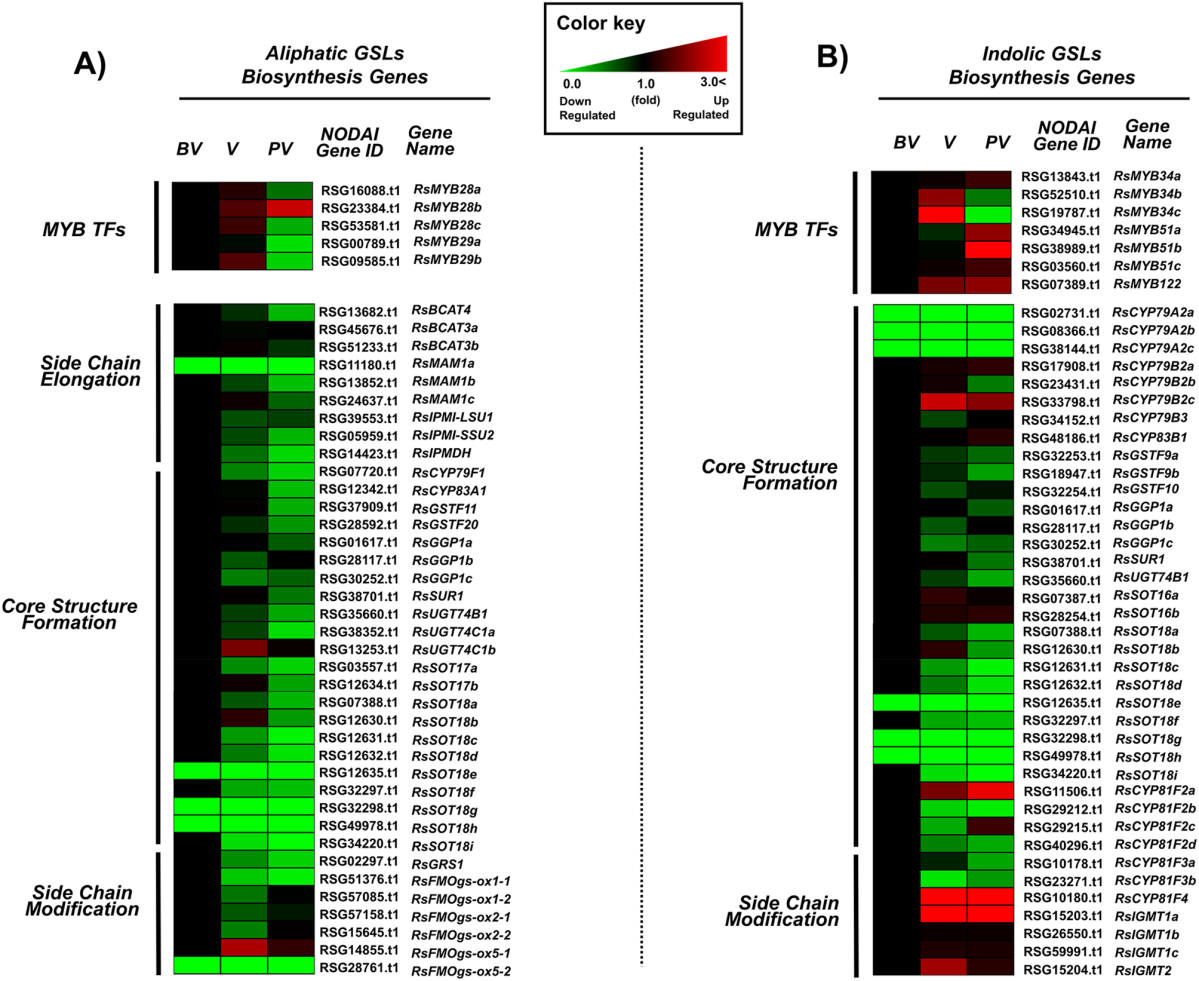
Heatmap of the transcriptional profiles of aliphatic (**A**) and indolic (**B**) GSLs pathway genes in radish (*R. sativus*) at different vernalization time points (*BV* before vernalization, *V* vernalization, *AV* after vernalization). The transcript value of each gene was normalized in comparison to the value before-vernalization (BV), which was set as 1.

In the present study, a total of 93 GSLs metabolic genes were identified in radish by blasting 63 *Arabidopsis* GSLs genes to the Nodai radish genome database (Supplementary Table S1). RNA-Seq data was analyzed to examine the expression profiles of these aliphatic and indolic GSLs biosynthetic genes before, during, and after vernalization, which were presented in a heatmap (Fig. 6). First, we examined the expression profiles of *MYB* transcription factors (TFs) regulating aliphatic and indolic GSLs biosynthesis (Fig. 5C,D). Homology information about *RsMYB* was adopted from radish genomic sequence that was previously reported^17^. The results showed that three *MYB28* homologs (*RsMYB28a*, RSG16088; *RsMYB28b*, RSG23384; *RsMYB28c*, RSG53581) and two *MYB29* homologs (*RsMYB29a*, RSG00789; *RsMYB29b*, RSG09585), which were involved in aliphatic GSLs biosynthesis, were identified in the radish genome. Among the homologs, two *RsMYB28* homologs (*RsMYB28a* and *RsMYB28c*) and two *RsMYB29* homologs (*RsMYB29a* and *RsMYB29b*) were highly expressed before vernalization, suggesting that they might be involved in aliphatic GSLs biosynthesis before vernalization (Fig. 5C). There was a slight increase in the expression of the homologs during vernalization; however, there was a significant decrease in their expression after vernalization (Figs. 5C, 6A), which was consistent with the results of the HPLC data (Supplementary Fig. S3C–S3D). Among the TFs, only *RsMYB28b* exhibited increasing pattern after vernalization; however, its expression was generally low, suggesting that *RsMYBb28b* might play a minor role during aliphatic GSLs biosynthesis (Fig. 5C).

Regarding *MYB* TFs regulating indolic GSLs biosynthesis, three *RsMYB34* homologs (*RsMYB34a*, RSG13843; *RsMYB34b*, RSG52510; *RsMYB34c*, RSG19787), three *RsMYB51* homologs (*RsMYB51a*, RSG34945; *RsMYB51b*, RSG38989; *RsMYB51c*, RSG03560), and one *RsMYB122* homolog (RSG07389) were identified in the radish genome database (Figs. 5D, 6B). Among them, *RsMYB34a*, *RsMYB51a*, and *RsMYB51b* were highly expressed before vernalization (Fig. 5D), suggesting that they might play a major role in indolic GSLs biosynthesis before vernalization. However, vernalization had varying effects on the expression profiles of the TFs (Figs. 5D, 6B). Additionally, the expression of *RsMYB51c* was low before, during, and after vernalization, indicating that it played minor role in indolic GSLs biosynthesis in radish. Furthermore, there was a significant increase in the expression of *RsMYB34a*, *RsMYB51a*, *RsMYB51b*, and *RsMYB122* after vernalization, in contrast to the results of the HPLC, which showed a decrease in indolic GSLs content of the plants after vernalization (Supplementary Fig. S3A and S3B). Although the radish *MYB* TFs were predicted to be homologs to *Arabidopsis MYB* TFs, they might be involved in other functions other than indolic GSLs biosynthesis after vernalization, which may the reason for the discrepancy between the results of the HPLC and RNA sequencing. Studies have shown that mutants of these *MYB* TFs were involved in auxin biosynthetic process^18–20^.

### Transcriptional profile of aliphatic GSLs metabolic genes

The aliphatic GSLs biosynthesis pathway is made up of three step-wise stages (Fig. 1). Genes involved in the three major stages, such as side-chain elongation, core structure synthesis, and secondary modification were identified before, during, and after vernalization. RNA-Seq data revealed that the expression of genes involved in aliphatic GSLs biosynthesis was significantly reduced by vernalization (Fig. 6A and Supplementary Fig. S5). However, there was a slight increase in the expression of two aliphatic GSLs biosynthesis-related genes (*RsUGT74C1b* and *RsFMOgs-OX5a*) during vernalization, but there was a decrease in their expression after vernalization (Fig. 6A).

In Fig. 4, increased conversion of GRE from GRH was detected during vernalization, suggesting that some FMOgs-OXs might play a role in this conversion. Confirming this, six FMOgs-OXs homologs were identified in the radish genome. Five genes out of six FMOgs-OXs displayed differential expression before, during, and after vernalization (Supplementary Fig. S5). Particularly, *FMOgs-ox5-1* and *FMOgs-ox2-2* exhibited the highest expression during vernalization, possibly contributing to the elevated amount of GRE during vernalization (Fig. 6A and Supplementary Fig. S5). These results indicated that the decreased expression of aliphatic GSLs biosynthesis-related genes during vernalization could be responsible for the corresponding decrease in the aliphatic GSLs content of the roots and leaves during vernalization.

### Transcriptional profile of biosynthetic genes in indolic GSLs pathways

Compared with aliphatic GSLs biosynthesis-related genes, the expression patterns of indolic GSLs metabolic genes were dynamic before, during, and after vernalization. For instance, the expression of several genes in core structure formation process were significantly reduced by vernalization (Fig. 6B), which was consistent with the reduced amounts of indolic GSLs compounds during vernalization. However, several genes (*RsCYP81F4* and *RsIGMT1a*) involved in the secondary modification process were activated by vernalization (Fig. 6B), which was not consistent with the results of HPLC (Supplementary Fig. S3A and S3B). This discrepancy could be as a result of low expression of several genes in core structure formation process, which may have contributed to the low indolic GSLs content of the samples regardless of the increase of the genes in the secondary modification stage. Another possibility is that there might be difference in the protein level and RNA transcript levels of genes involved in the secondary modification stage of indolic GSLs pathway. That is, although the genes were highly expressed, the levels of corresponding proteins could be low or unstable during vernalization.

### Expression of *RsGRS1* along vernalization time points

**Figure 7:**
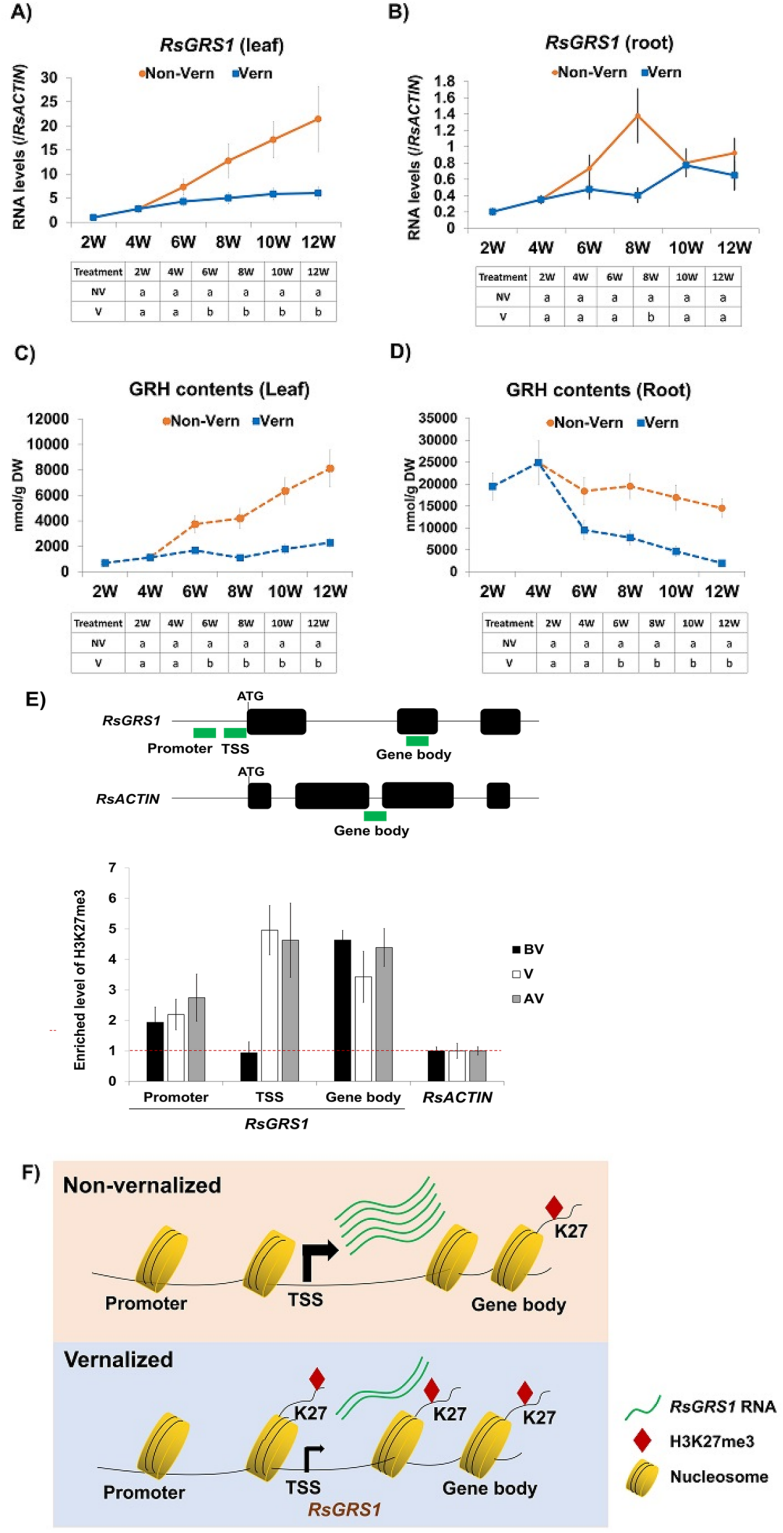
Transcriptional profiles of *RsGRS1* gene in relation to the corresponding GRH contents of the leaves and roots of vernalized and non-vernalized *R. sativus*. The expression profiles of *RsGRS1* in the leaves (**A**) and roots (**B**) were represented with solid lines, while the content of corresponding GRH in the lea (**C**) and roots (**D**) were represented with dashed lines. The expression of *RsGRS1* was normalized to the value of 2-weeks-old leaf sample (value = 1). (**E**) Result of ChIP-qPCR for genomic region of *RsGRS1*. Upper panel: Three PCR amplicons (promoter, TSS, and gene body) spanning promoter, transcription start site (TSS), and gene body were adopted in the qPCR (upper panel). In addition, amplicon amplifying gene body region of *RsACTIN,* a H3K27me3-depleted gene was used as a control. Bottom panel: Result of ChIP-qPCR. Precipitated and input DNA from ChIP assay using antibody against H3K27me3 histone mark were used for qPCR, then the relative enrichment was determined by comparing to the values of *RsACTIN* (set as 1) along three time points (BV, V, and AV). (**F**) Schematic illustration of epigenetic suppression of *RsGRS1* during vernalization via a repressive histone mark, H3K27me3. In non-vernalized condition (upper panel), H3K27me3 is poorly enriched at promoter and TSS site of *RsGRS1*, thus resulting in high transcription activity. In contrast, H3K27me3 was highly enriched at the TSS region of *RsGRS1* during vernalization (lower panel), decreasing transcriptional activity. Data were presented as mean ± standard deviation (SD) (n = 3). Statistically significant differences were determined by one-way ANOVA and Tukey’s post hoc test (*p* < 0.05).

As shown in Fig. 3, GRH constitutes the highest proportion of aliphatic GSLs in radish plant, a unique aliphatic GSLs compositional profile compared with other plants of family Brassicaceae (Nugroho et al., 2019). Recently, a gene responsible for predominant GRH in radish was identified and named as radish *GLUCORAPHASATIN SYNTHASE 1* (*RsGRS1*, RSG02297). *RsGRS1* encodes 2-oxoglutarate-dependent dioxygenase and catalyzes the conversion of GER to GRH^12^. There was an increase in the expression of *RsGRS1* in both the leaves and roots of non-vernalized plants with increase in age (Fig. 7A,B). However, there was no marked increase in the expression of *RsGRS1* in the leaves and roots of vernalized plants. Additionally, the expression of *RsGRS1* was significantly higher in the leaves than in the roots of both non-vernalized and vernalized plants (Fig. 7A,B), suggesting a higher activity of *RsGRS1* in the leaves of radish than in the roots. Since *RsGRS1* is involved in the conversion of GER to GRH, we determined the GRH contents of the leaves and roots before, during, and after vernalization (Fig. 7C,D), and found that the GRH content of the leaves of non-vernalized plants increased with plant age, confirming the results of the transcriptome analysis (Fig. 7C). In contrast, the GRH content of the roots of non-vernalized and vernalized plants decreased with increase in plant age (Fig. 7D). These results indicated that vernalization decreased the expression of *RsGRS1* and its corresponding metabolic product (GRH) in radish.

### Epigenetic suppression of *RsGRS1* by vernalization

Vernalization triggers flowering in *Arabidopsis* and in several plants of family *Brassicaceae* by inhibiting the expression of *FLOWERING LOCUS C* (*FLC*), a potent floral repressor gene, through epigenetic histone modification^21,22^. Particularly, a repressive histone mark, tri-methylation at lysine 27 residue of histone H3 (H3K27em3) is highly enriched at *FLC* chromatin during vernalization, which stably inhibits the expression of *FLC*. Since *RsGRS1* was suppressed during vernalization, we investigated whether vernalization-mediated suppression of *RsGRS1* is through epigenetic histone modification. Therefore, we performed a chromatin immunoprecipitation (ChIP)-qPCR assay using an antibody against H3K27me3 (Fig. 7E). Three PCR amplicons spanning the entire *RsGRS1* genomic region (promoter, TSS, and gene body) were used for ChIP-qPCR analysis (Fig. 7E, upper panel). In addition, for relative quantification of enriched H3K27me3, *RsACTIN* (Rs281010) was used as a negative control because housekeeping genes such as *ACTIN, UBQ10,* and *PP2A* genes are usually deprived of H3K27me3 in *Arabidopsis* model plant. In ChIP-qPCR analysis, we found that *RsACTIN* was merely enriched with H3K27me3 (Fig. 7E, bottom panel). Meanwhile, regarding the *RsGRS1* genomic region, we noticed that gene body region of *RsGRS1* was consistently enriched with H3K27me3 before, during and after vernalization. In contrast, H3K27me3 at TSS (transcription start site) of *RsGRS1* was lowly detected before vernalization, but significantly increased during and after vernalization, indicating that the expression of *RsGRS1* was suppressed by vernalization. Additionally, enrichment of H3K27me3 at TSS region is critical for inhibiting *RsGRS1*. The promoter region of *RsGRS1* was not significantly enriched before, during, and after vernalization. These results were consistent with previous reports that H3K27me3 are highly enriched at gene coding region including TSS^15,23–25^. Overall, *RsGRS1* experienced epigenetic suppression during vernalization via enrichment of H3K27me3 at TSS region (Fig. 7F), which resulted in reduced concentrations of GRH in both the leaf and root tissues of radish after vernalization.

**Figure 8:**
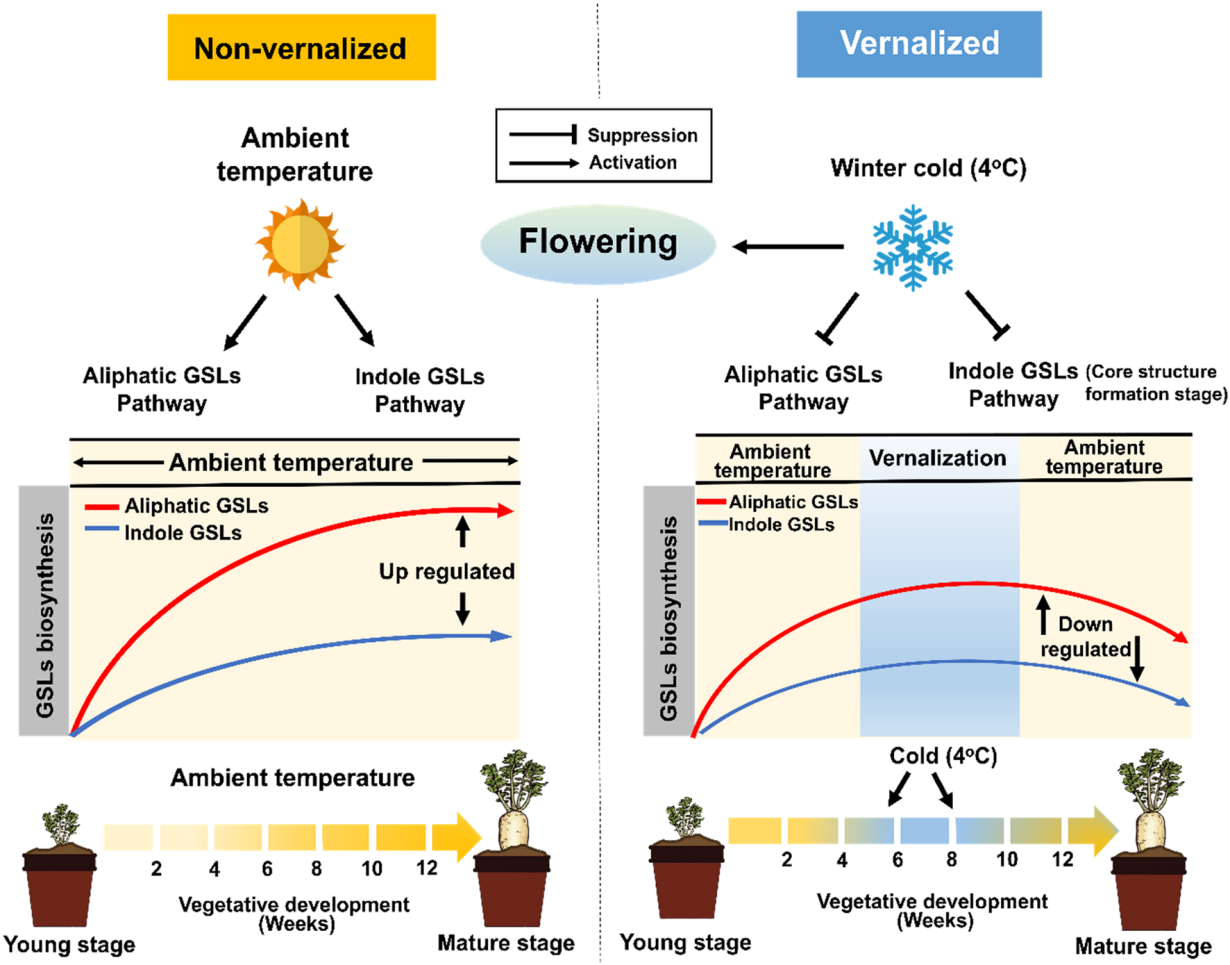
Schematic illustration of vernalization-mediated suppression of GSLs pathway genes and corresponding GSLs compounds in radish plants. The aliphatic and indolic GSLs contents non-vernalized radish plants increased with plant age (2–12 weeks). Vernalization suppressed the biosynthesis of aliphatic and indolic GSLs by suppressing the expression of GSLs pathway genes at the transcriptional level.

This study demonstrated that GSLs biosynthesis increased along vegetative developmental stages (2-week ~ 12-week), possibly to cope with abiotic and biotic stresses (i.e. insect and pathogen attacks) in warm temperature (Fig. 8). Meanwhile, vernalization suppressed biosynthesis of both aliphatic and indolic GSLs at the transcriptional level. Recently, it was reported that plants need sugar to keep the turgidity of cell from the chilling injury^26^. Thus, we speculate that plants reduce biosynthesis of glucose-containing GSLs, instead increase synthesis of the sugars to cope with the cold stress during vernalization. This hypothesis needs to be investigated.

## Discussion

Vernalization accelerates plant development from vegetative to reproductive stage, and this process is necessary for the optimal flowering of several plants of the family Brassicaceae. The complexity of epigenetic mechanism including histone modifications control floral transition by vernalization. The mechanisms of vernalization have been extensively studied in *Arabidopsis thaliana* and in some plants of family Brassicaceae^13,21,27–29^. However, the mechanisms underlying physiological and biological changes in plants during vernalization remains unclear. The transition from vegetative stage to reproductive stage in plants^30,31^ is accompanied by considerable changes in the secondary metabolites profiles of plants, such as changes in GSLs concentration. GSLs biosynthesis is modulated at different developmental stages in radish^11,16,32^. In the present study, HPLC, RNA-seq, and RT-qPCR showed that vernalization reduced the GSLs content of the leaves and roots of radish plants compared with that of non-vernalized plants.

Particularly, the results of the study showed that GRH was the most abundant aliphatic GSLs in the roots and leaves of 2–12-weeks-old plants (Fig. 3). GSLs and some of their hydrolyzed products are known to exert anti-pathogenic activity during biotic stresses as well as anti-carcinogenic effects against human cancer cells. For instance, glucoraphanin (GRA) and its hydrolyzed product, sulforaphane (SFA), which are the primary GSLs in broccoli, have been reported to possess anticarcinogenic effect^33^. However, whether GRH and its hydrolyzed products, which were detected in radish, possess anti-pathogenic effects against insect or pathogens and their roles in human health is subject to further studies.

Interestingly, it was observed that although the expression of *RsGRS1* was higher in the leaves than in the roots, concentration of GRH was significantly higher in the roots than in the leaves of both non-vernalized and vernalized plants (Fig. 7C,D). Previous studies suggested that GRH biosynthesis occurs in leaf tissue, but is translocated and accumulated in the root tissue in radish^11,12,34^. Based on this, we speculated that although *RsGRS1* was highly activated to synthesize GRH in the leaf tissue, the synthesized GRH was translocated to the root tissue, possibly by glucosinolates transporters (GTRs), resulting in high amounts of GRH in root of radish. However, this hypothesis is subject to further studies.

In *Arabidopsis*, vernalization increases flowering by inhibiting the expression of *FLC* gene, a potent floral repressor^27^. Suppression of *FLC* by vernalization is accompanied with epigenetic histone modifications, particularly deposition of repressive histone marks like H3K27me3 on *FLC* chromatin during vernalization.

A group of R2R3-MYB family transcription factors (TFs) are involved in the biosynthesis of aliphatic and indolic GSLs^35–37^. It has been previously reported that prolong exposure to cold conditions suppressed the expression of *MYB28* and *MYB29* in *Brassica rapa*^38^. Similarly, in the present study, the expression profiles of homologs of *RsMYB28* and *RsMYB29* (other than *RsMYB28b*) controlling aliphatic GSLs biosynthesis were significantly downregulated during and after vernalization compared with that of non-vernalized plants (Figs. 6A, 5C,D). As shown in Fig. 7E that *RsGRS1* is suppressed during vernalization via epigenetic modification, it is possible that vernalization might also target these *RsMYB* TFs as well as other GSLs pathway genes to modulate aliphatic GSLs biosynthesis. Therefore, future studies should clarify whether the *RsMYB* TFs and other GSLs biosynthetic genes undergo epigenetic modification(s) during vernalization.

Consistent with the decrease in the expression of *RsMYB28s* and *RsMYB29s* TFs in aliphatic GSLs pathway during vernalization, the expression profiles of most genes related to GSLs biosynthesis were significantly downregulated in the aliphatic GSLs pathway after vernalization, (Fig. 6A)*.* Although, the upstream regulator(s) responsible for activating these *MYB* TFs were not identified in the present study, *VERNALIZATION INSENSITIVE 3* (*VIN3*) gene was identified in Arabidopsis mutant as a upstream promoter of these *MYB* TFs^39,40^. VIN3 encodes a PHD-finger domain protein and play a pivotal role in vernalization response in Arabidopsis^27,28^. Therefore, future studies should attempt to identify upstream factor(s) controlling these GSLs-related MYB TFs in radish.

There was a decrease in the GRH content of the plants and an increase in the GRE content during vernalization (Fig. 3A), which could be due to the conversion of GRH to GRE during vernalization. Studies have shown that FMOgs-OXs catalyzes the conversion of methylthioalkyl (GRH) to methylsulphinyl butyl (GRE)^41,42^. Till date, six *FMOgs-OXs* homologs (*FMOgs-OX1-1, FMOgs-OX1-2, FMOgs-OX2-1, FMOgs-OX2-2, FMOgs-OX5-1,* and *FMOgs-OX5-2*) have been identified in radish genome (Supplementary Table S1), among which five were expressed and quantified in the presents study (Fig. 6A and Supplementary Fig. S6). Vernalization decreased the expression of most *FMOgs-OXs* except *FMOgs-OX5-1*. In contrast, there was an increase in the expression of *FMOgs-OX5-1* during vernalization (Fig. 6A and Supplementary Fig. S6). Considering that the concentration of GRE was gradually increased during vernalization, it is possibly that *FMOgs-OX5-1* was responsible for the increase. Moreover, since GRE can be further converted to isothiocyanate which exerts defense against biotic stresses (i.e. insect, herbivore, and pathogen attacks)^43–45^, increased GRE might contribute to the establishment of preliminary defense system. However, the functions of *FMOgs-OX5-1* in vernalization response is subject to further studies.

Although vernalization promotes flowering, it is considered a cold stress. It has been reported that short-term cold stress can reduce the total GSLs content of kale^46^. Similarly, the results of the present study showed that vernalization decreased the total GSLs content of radish, indicating that cold stress whether short- or long-term could cause a decrease in GSLs content of plants. Plants might use their limited energy and resources to cope with cold stress, at least partly by decreasing the production of GSLs, which is necessary for defense against insect or pathogens. It is reasonable speculation because most of insects and pathogens are not active during winter cold season. This indicate that GSLs are not involved in cold stress response in radish plants.

In conclusion, we reported that vernalization reduced the total GSLs contents of the roots and leaves of radish. Additionally, vernalization suppressed the biosynthesis of both aliphatic and indolic GSLs via the modulation of the transcription of GSLs metabolic genes.

## Materials and methods

### Plant growth and vernalization treatment

The seeds of radish cv. Taebaek was kindly gifted from Syngenta Korea Co in South Korea. Seeds were germinated on wet filter paper and incubated in the dark at 28 ± 1 °C overnight. Germinated seedlings were sown in pots in a greenhouse for vegetative growth. Four weeks after planting, the seedling was separated into two treatment groups: vernalized (V; cold temperature) and non-vernalized control (NV; greenhouse-ambient temperature) groups. For the vernalized group, the seedlings were transferred and grown in a cold room (temperature, 4 ± 1 °C; relative humidity, 80 ± 10%) under an 8-h light/16-h dark photoperiod (sodium light) for 4 weeks. For the non-vernalized control group, the seedlings were grown in the greenhouse at 22 ± 1 °C during the night and 28 ± 1 °C during the day. After 4 weeks of cold treatment, the vernalized plants (8-weeks old) were transferred back into the greenhouse and grown under the same condition with the non-vernalized plants. Leaf and root samples were collected from three biological replicates of both vernalized and non-vernalized plants at 2, 4, 6, 8, 10, and 12 weeks of age and stored in a freezer at − 80 °C.

### Extraction and analysis of GSLs

Desulfo-GSLs (DS-GSLs) was extracted as described previously^11,47^. Briefly, fresh plant tissues were lyophilized using vacuum freeze dryer (Ilshin Lab, South Korea) and ground into fine powder for GSLs analysis. The lyophilized samples were incubated with 70% MeOH (10 ml) at 70 °C for 10 min to inhibit myrosinase activity. Thereafter, the extract was transferred into a polypropylene column (Thermo Scientific, USA) and allowed to react with 11.25 units of sulfatase for 12 h at 37 °C. After incubation, column was eluted gradually with 1.5 ml deionized water and evaporated by speed vacuum afterwards. The DS-GSLs was redissolved in 1 ml HPLC water (Fisher scientific, USA), filtered using 0.45 μm PVDF membrane (Biofact, Korea), and transferred into HPLC vial.

The DS-GSLs were separated and analyzed as previously described using Dionex Ultimate 3000 ultra-high performance liquid chromatography (U-HPLC) systems (Thermo Scientific, USA)^11,47^. The DS-GSLs were separated on a C18 reverse phase column (Zorbax XDB-C18, 4.6 × 250 mm^2^, 5 μm particle size, Agilent, USA) with a water (Fisher Scientific, USA) and acetonitrile (Honeywell, USA) gradient system. Samples (20 µL) of DS-GSLs were injected at flow rate 1.0 mL min^−1^ and analyzed using diode array detector at 229 nm. Peaks representing different GSLs compounds were identified using standard compounds (Sigma-Aldrich, USA), and sinigrin was used for relative quantification as previously reported^16^. The contents were analyzed independently with three biological replicates and presented as micromoles per kilogram dry weight (nmol/g DW).

### Total RNA extraction and RNA Sequencing

Total RNAs from leaf and root tissues of radish were isolated using TRIzol reagent (Invitrogen, USA), according to the manufacturer’s instructions. Total RNA was treated with DNaseI (Sigma-Aldrich, USA) to remove residual genomic DNA, and purified using NucleoSpin RNA Clean-up Kit (Macherey–Nagel, Germany) to remove unwanted materials. For the analysis, samples from 4-, 8-, and 10-weeks old radish were classified as before vernalization (BV), vernalization (V), after vernalization (AV), respectively.

### RNA-Seq library construction and Illumina sequencing

For each time point (BV, V, and AV), three biological replicates were collected and ground in liquid nitrogen for total RNA extraction. Total RNAs were isolated using the RNeasy mini kit (Qiagen, USA). Illumina cDNA libraries were constructed using TruSeq Stranded mRNA LT Sample Prep Kits (Illumina, Inc., USA), according to the manufacturer’s instructions. Sequencing of the cDNA libraries was performed by Macrogen Inc. (Republic of Korea) on an Illumina platform using Illumina NovaSeq6000 system.

### Sequence alignment and analysis

Prior to alignments of RNA-seq reads to the Radish reference genome (http://www.nodai-genome-d.org/), quality of raw reads was evaluated with the FastQC software (http://www.bioinformatics.babraham.ac.uk/projects/fastqc/) for quality assessment^48,49^. Based on the FastQC results, individual reads were trimmed and quality-filtered using fastx-trimmer software (http://hannonlab.cshl.edu/fastx_toolkit/)^50^. Trimmed reads with more than 95% portion Q > 30 were aligned using Tophat2 software with default parameters^51^. Aligned reads were converted to digital counts using HTseq-count and further analyzed to identify differentially expressed genes (DEGs) using edgeR^52^. Hierarchical clustering analysis of DEGs was performed using Python-based in-house script. Multi-dimensional Scaling (MDS) plot and correlation heatmap were generated using R software (ver. 3.6.0) (https://www.r-project.org/). Venn diagram data was generated using VENNY webtool (version 2.1.0) (https://bioinfogp.cnb.csic.es/tools/venny/)^53^. Heatmap analysis was performed using multi experiment viewer (MEV) program (ver 4.9.0)^54^.

### Radish glucosinolates (GSLs) biosynthetic genes and related MYB transcription factors

To get information on radish genes involved in the glucosinolates (GSLs) biosynthesis and related MYB transcription factors, we first got sequence information of Arabidopsis GSL pathway genes from Arabidopsis genome database (www.arabidopsis.org). A total of 55 *Arabidopsis* GSL pathway genes including MYB transcription factors were collected and used to obtain radish homologs by the blast search program in a radish genome database (www/nodai-genome-d.org/). A total of 93 radish GSL pathway genes were identified and listed in the Supplementary Table S1.

### Quantitative RT-PCR

Gene expression analysis was performed as previously described^11^. The first-strand cDNA was synthesized using AccuPower RT-Premix (Bioneer, Korea), according to the manufacturer’s instruction. RT-qPCR was determined on ABI PRISM 7500 Real-Time PCR system (Life Technologies, USA). Gene expression analysis was performed using Accupower 2 × Greenstar qPCR Master Mix (Bioneer, Korea) with three technical replicates. The relative expression of each gene was obtained by normalization to the *ACTIN* reference gene. Primer pairs used in this study are listed described in Supplementary Table S4.

### Chromatin immunoprecipitation (ChIP)-qPCR analysis

Seedlings were cross-linked with 1% formaldehyde solution under vacuum for 25 min, then terminated by addition of 0.125 M glycine. Cross-linked seedlings were dried and then frozen in liquid nitrogen. ChIP experiment was performed as previously reported^51^. Ten micrograms of monoclonal antibody against H3K27me3 histone mark (ab6002, Abcam, United Kingdom) were used for individual ChIP sample. Aliquots of immunoprecipitated and eluted input DNAs were used for qPCR analysis.

### Statistical analysis

Statistical analysis of data obtained in the study was performed using SAS statistical software v. 9.4 (SAS Institute Inc., Carry, NC, USA). Statistically significant differences were determined by one-way ANOVA and Tukey’s post hoc test (*p* < 0.05).

## Supplementary Information

Supplementary Information.

## Data Availability

The transcriptome data analyzed in this study can be found in the Gene Expression Omnibus (GEO accession number GSE182885).

## Abbreviations

PGT: Progoitrin
GER: Glucoerucin
GRH: Glucoraphasatin
GRE: Glucoraphenin
GRA: Glucoraphanin
SFA: Sulforaphane
TFs: Transcription factors family
GRS1: Glucoraphasatin synthase 1
FLC: Flowering locus C
*FMOgs-OXs*: Flavin monooxygenase
UHPLC: Ultra-high performance liquid chromatography
RT-qPCR: Quantitative reverse transcription PCR
