# Supplementary material for: Transcriptomic and metabolic analyses revealed the modulatory effect of vernalization on glucosinolate metabolism in radish (Raphanus sativus L.)
Source: Sci Rep. 2021 Dec 15;11:24023. doi: 10.1038/s41598-021-03557-5 (PMC8674254; doi:10.1038/s41598-021-03557-5)
Supplement: Supplementary file 1 — Supplementary Information. [file 41598_2021_3557_MOESM1_ESM.docx]

***Supplementary Information***

Article title: **Transcriptomic and metabolic analyses revealed the modulatory effect of vernalization on glucosinolate metabolism in radish (*Raphanus sativus* L.)**

Authors: Adji Baskoro Dwi Nugroho^1^, Sangwoo Lee^1^, Aditya Nurmalita Pervitasari^1^, Heewon Moon^1^, Dasom Choi^1^, Jongkee Kim^1^, Dong-Hwan Kim^1,2^*

The following Supplementary Information is available for this article:

**Supplementary Figures**

**Supplementary Fig. S1** U-HPLC chromatogram of GSLs in leaves and roots of vernalized and non-vernalized radish. 1, progoitrin (PGT); 2, glucoraphanin (GRE); 3, 4-hydroxyglucobrassicin (4-HGB); 4, glucoerucin (GER); 5, glucoraphasatin (GRH); 6, glucobrassicin (GBS); 7, 4-methoxyglucobrassicin (4-MTGB).

**Supplementary Fig. S2** U-HPLC chromatogram of glucosinolates (GSLs) standard compounds. 1, Progoitrin (PGT); 2, glucoraphanin (GRA); 3, glucoraphanin (GRE); 4, sinigrin (SIN); 5, glucoalyssin (GAS); 6, gluconapin (GNP); 7, 4-Hydroxyglucobrassicin (4-HGB); 8, glucobrassicanapin (GBN); 9, glucoerucin (GER); 10, glucoraphasatin (GRH); 11, glucobrassicin (GBS); 12, 4-methoxyglucobrassicin (4-MTGB); 13, gluconasturtiin (GNT); 13, neoglucobrassicin (NGB).

**Supplementary Fig.** **S3** Comparison of the total GSLs content of the leaves (A) and roots (B) of vernalized and non-vernalized radish plants. Data were presented as mean ± standard deviation (SD) (n=3). Statistically significant differences were determined by one-way ANOVA and Tukey’s post hoc test (*p* < 0.05).

**Supplementary Fig.** **S4** Dynamic changes in the aliphatic and indolic GSLs contents of the leaves and roots of vernalized and non-vernalized radish plants. Data were presented as mean ± standard deviation (SD) (n=3). Statistically significant differences were determined by one-way ANOVA and Tukey’s post hoc test (*p* < 0.05).

**Supplementary Fig. S5** Profiling of RNA-Seq libraries. **(A)** MDS plot analysis for nine RNA-seq libraries. The color of square, circle, triangle corresponds to different vernalization time point (black: non-vernalized, green: vernalized, red: after vernalized). Multi-dimensional Scaling (MDS) plot was generated using R software (ver 3.6.0) (<https://www.r-project.org/>). **(B)** PlotSmear analysis showing differentially expressed genes. X -axis is the log2 value of read counts per million (CPM). Y -axis is log2 fold change (FC). Black dots represent the genes with no significant differential expression. Red dots represent significantly differentially expressed genes. PlotSmear was generated using R software (ver. 3.6.0) (<https://www.r-project.org/>).

**Supplementary Fig. S6** Quantification of RNA-seq reads counts in five *RsFMOgs-oxs (RsFMOgs-ox1-1, RsFMOgs-ox1-2, RsFMOgs-ox2-1, RsFMOgs-ox2-2,* and *RsFMOgs-ox5-1)* among three different time point. Data were presented as mean ± standard deviation (SD) (n=3). Statistically significant differences were determined by one-way ANOVA and Tukey’s post hoc test (*p* < 0.05).

**Supplementary Tables**

**Supplementary Table S1** Total 93 GSL metabolic pathway related genes in radish.

**Supplementary Table S2** Expression profiles of 93 GSL metabolic genes in non-vernalized and vernalized radish

**Supplementary Table S3** Total reads and mapped reads on the radish reference genome

**Supplementary Table S4** List of primers used in this study

**Supplementary Table S5** List of standard compounds of aliphatic GSL that used in this study

**Supplementary Fig. S1**


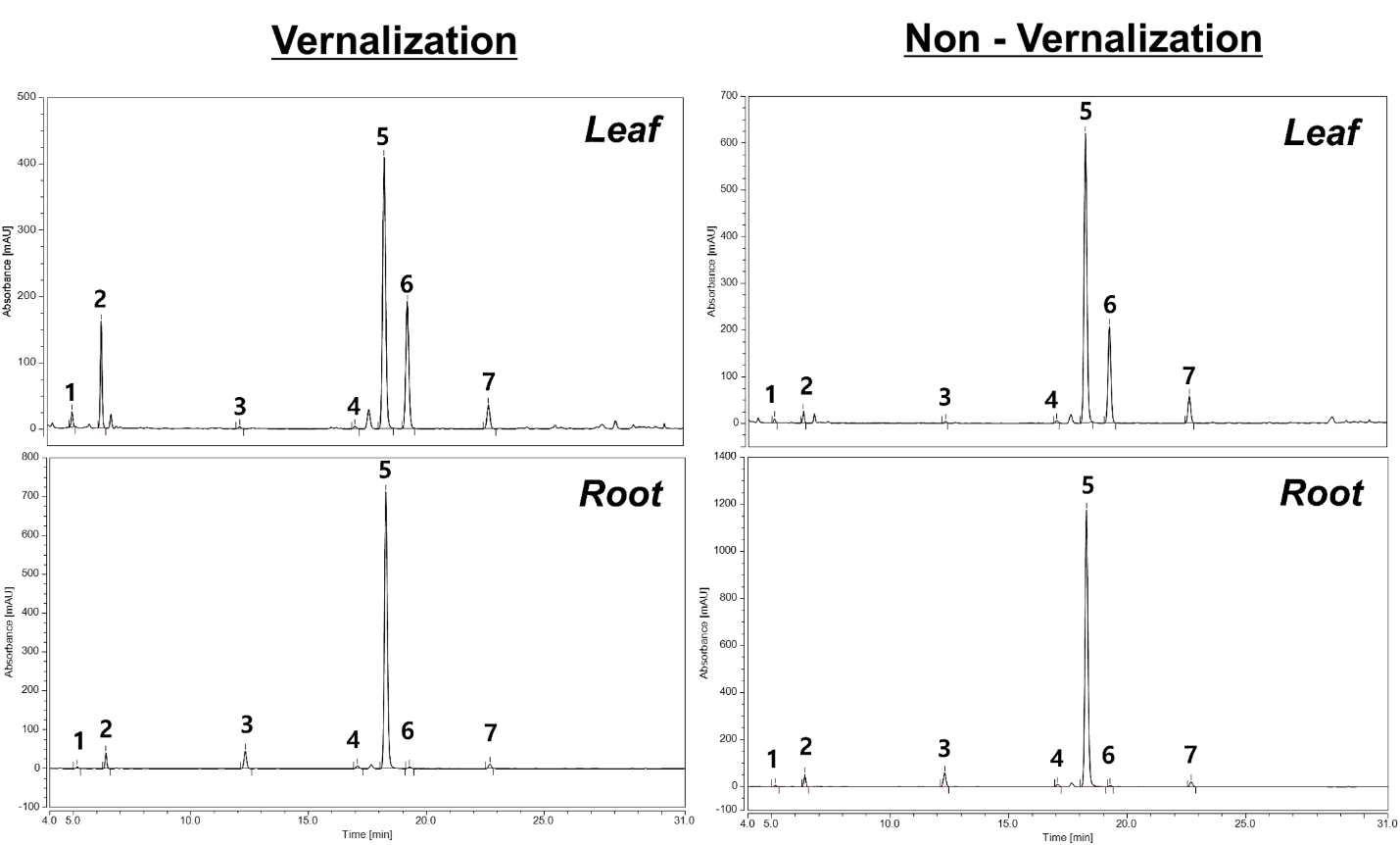


**Supplementary Fig. S1** U-HPLC chromatogram of GSLs in leaves and roots of vernalized and non-vernalized radish. 1, progoitrin (PGT); 2, glucoraphanin (GRE); 3, 4-hydroxyglucobrassicin (4-HGB); 4, glucoerucin (GER); 5, glucoraphasatin (GRH); 6, glucobrassicin (GBS); 7, 4-methoxyglucobrassicin (4-MTGB).

**Supplementary Fig. S2**

**
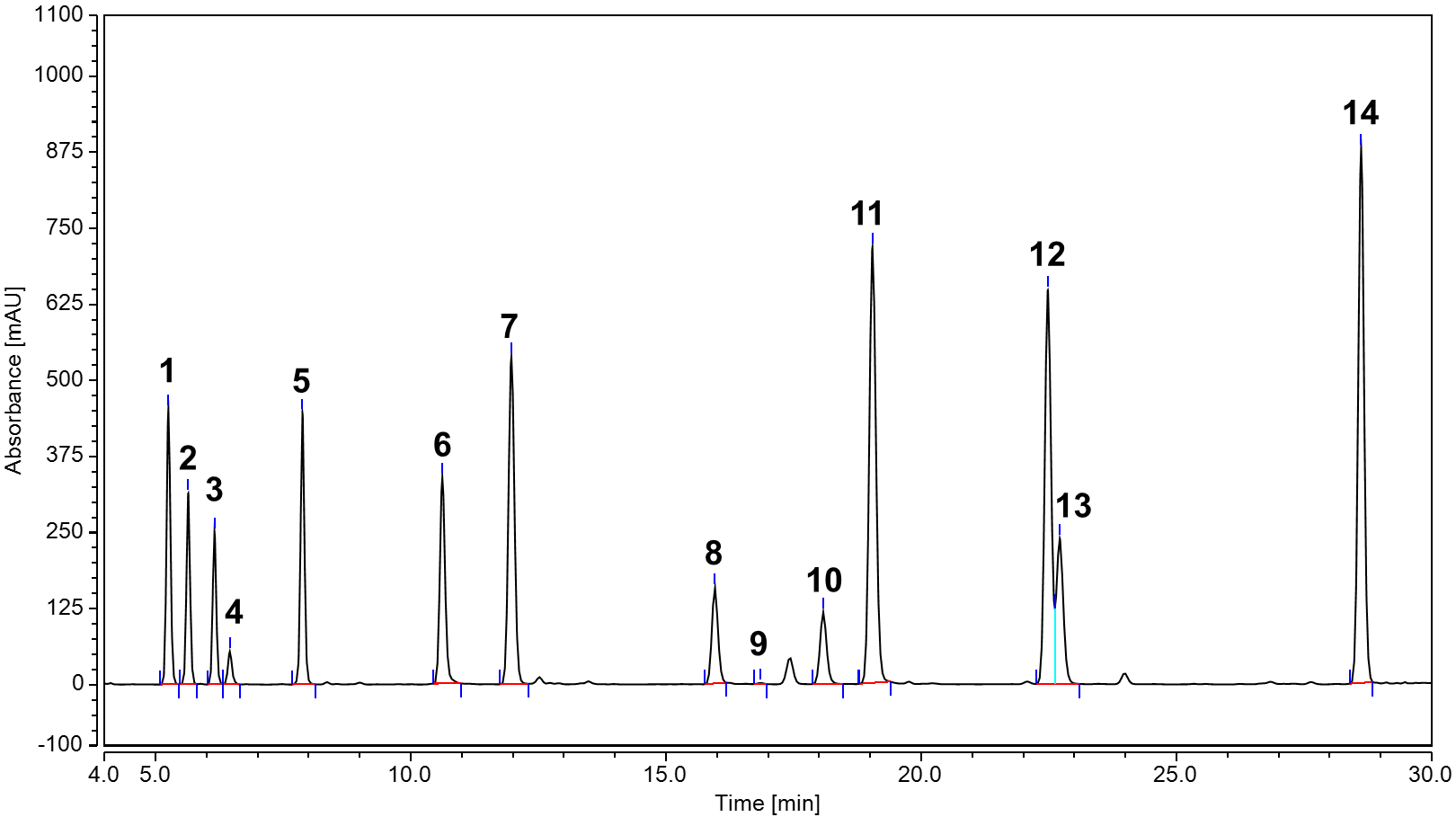
**

**Supplementary Fig. S2** U-HPLC chromatogram of glucosinolates (GSLs) standard compounds. 1, Progoitrin (PGT); 2, glucoraphanin (GRA); 3, glucoraphanin (GRE); 4, sinigrin (SIN); 5, glucoalyssin (GAS); 6, gluconapin (GNP); 7, 4-Hydroxyglucobrassicin (4-HGB); 8, glucobrassicanapin (GBN); 9, glucoerucin (GER); 10, glucoraphasatin (GRH); 11, glucobrassicin (GBS); 12, 4-methoxyglucobrassicin (4-MTGB); 13, gluconasturtiin (GNT); 13, neoglucobrassicin (NGB).

**Supplementary Fig. S3**


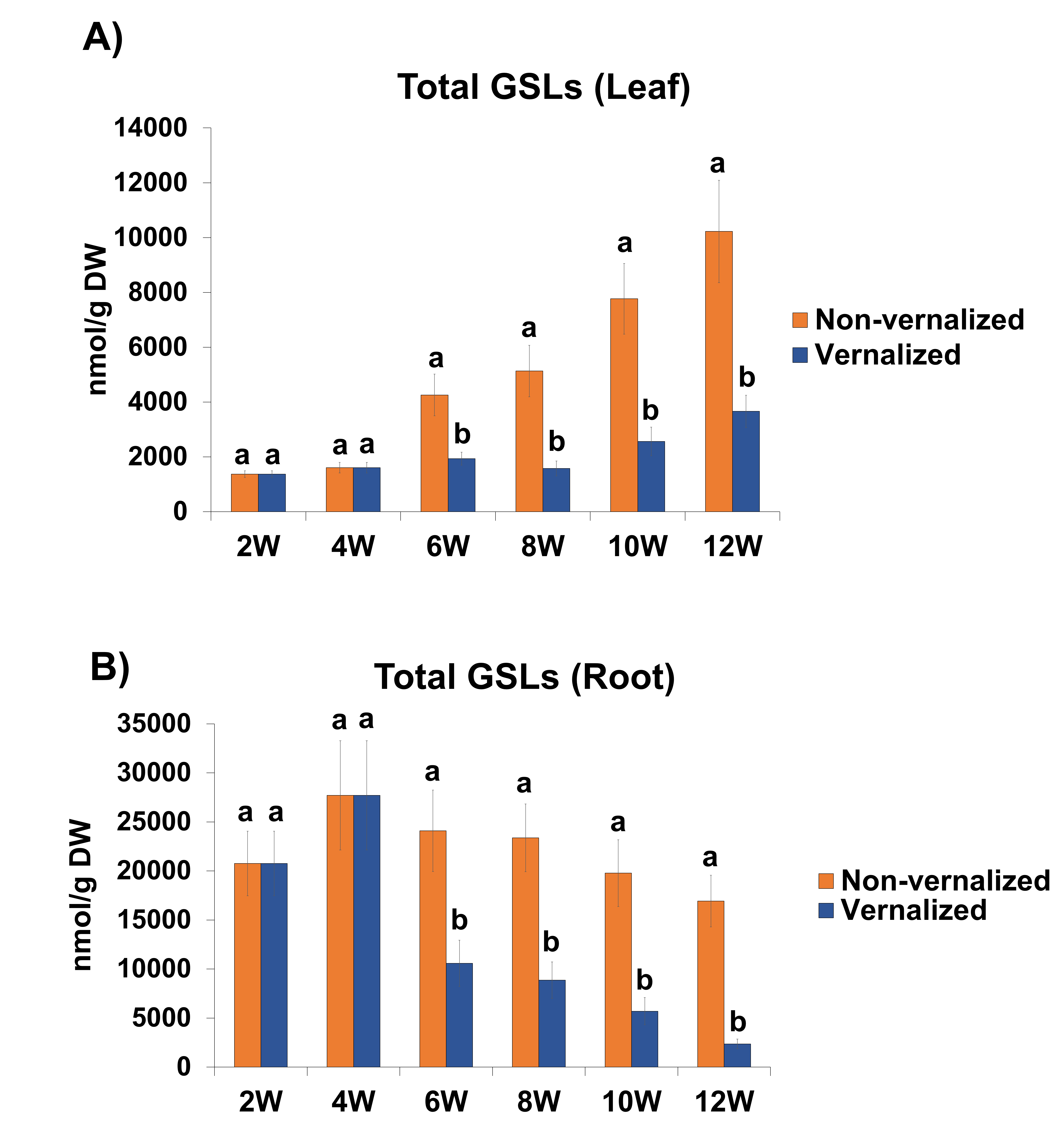


**Supplementary Fig. S3** Comparison of the total GSLs contents of the leaves **(A)** and roots **(B)** of vernalized and non-vernalized *R. sativus*. Data were presented as mean ± standard deviation (SD) (n=3). Statistically significant differences were determined by one-way ANOVA and Tukey’s post hoc test (*p* < 0.05).

**Supplementary Fig. S4**


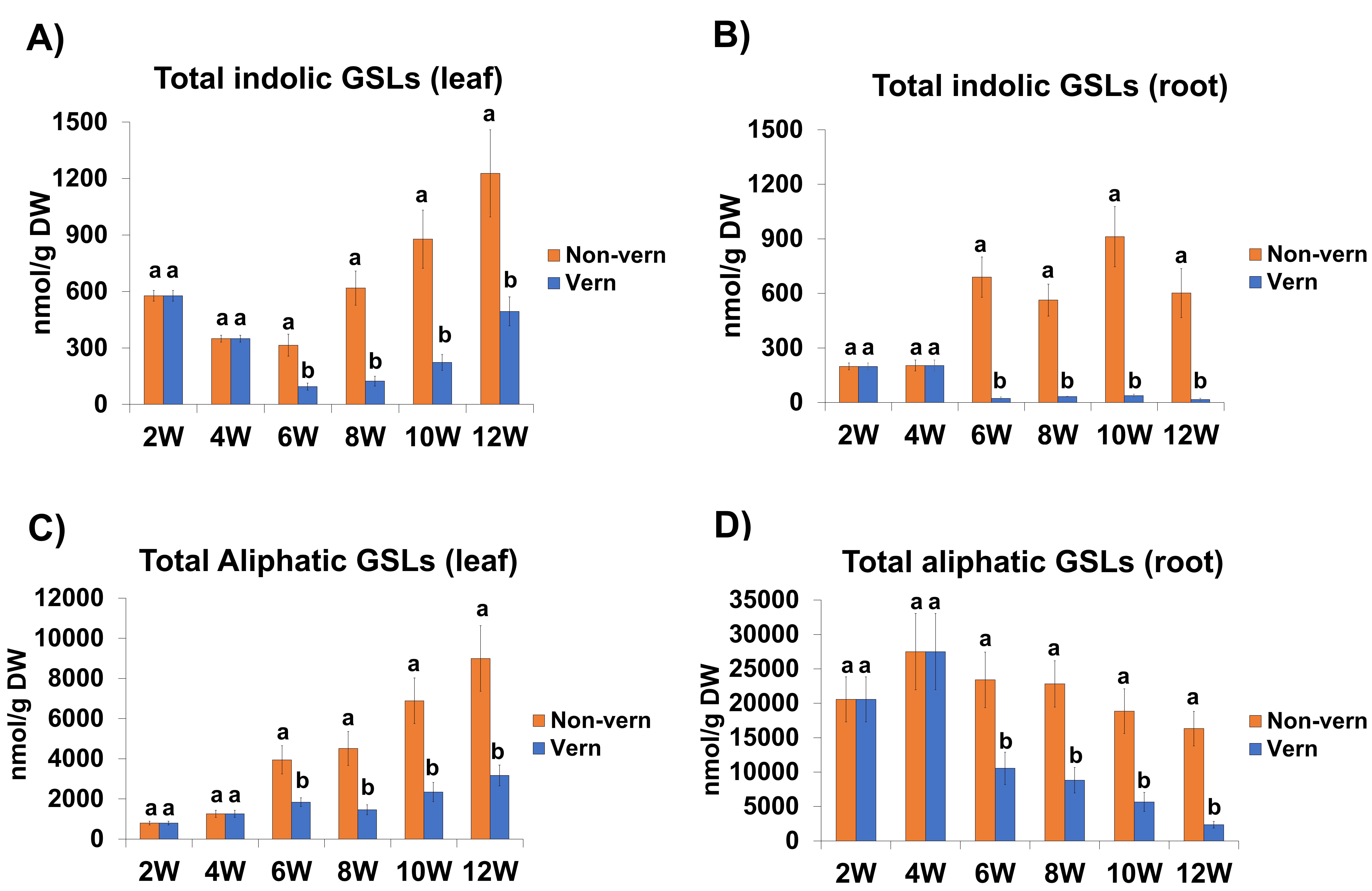


**Supplementary Fig. S4** Dynamic changes in the aliphatic and indolic GSLs contents of the leaves and roots of vernalized and non-vernalized radish plants. Data were presented as mean ± standard deviation (SD) (n=3). Statistically significant differences were determined by one-way ANOVA and Tukey’s post hoc test (*p* < 0.05).

**Supplementary Fig. S5**

**
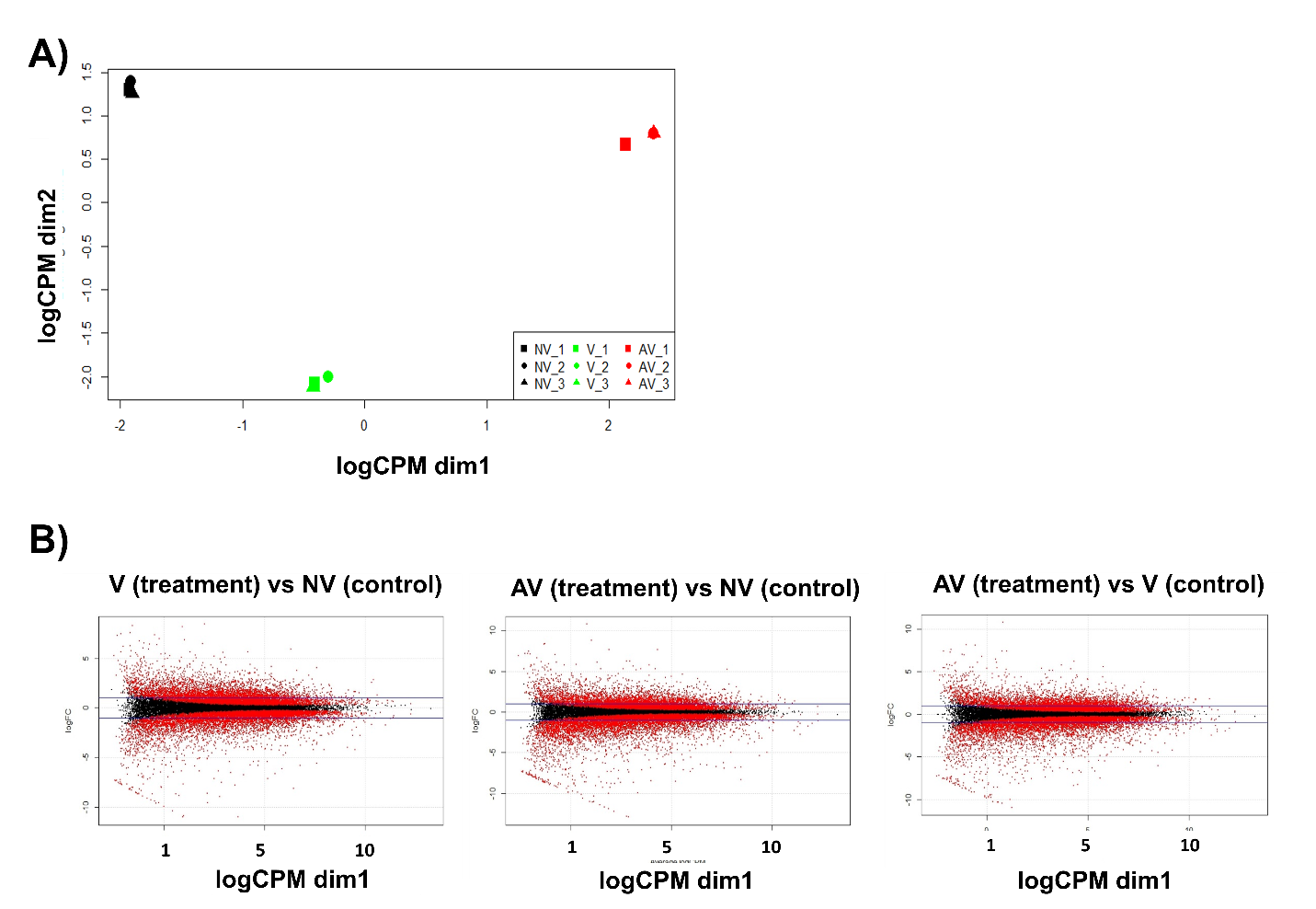
**

**Supplementary Fig. S5** Profiling of RNA-Seq libraries. **(A)** MDS plot analysis for nine RNA-seq libraries. The color of square, circle, triangle corresponds to different vernalization time point (black: non-vernalized, green: vernalized, red: after vernalized). Multi-dimensional Scaling (MDS) plot was generated using R software (ver 3.6.0) (<https://www.r-project.org/>). **(B)** PlotSmear analysis showing differentially expressed genes. X -axis is the log2 value of read counts per million (CPM). Y -axis is log2 fold change (FC). Black dots represent the genes with no significant differential expression. Red dots represent significantly differentially expressed genes. PlotSmear was generated using R software (ver. 3.6.0) (<https://www.r-project.org/>).

**Supplementary Fig. S6**

**
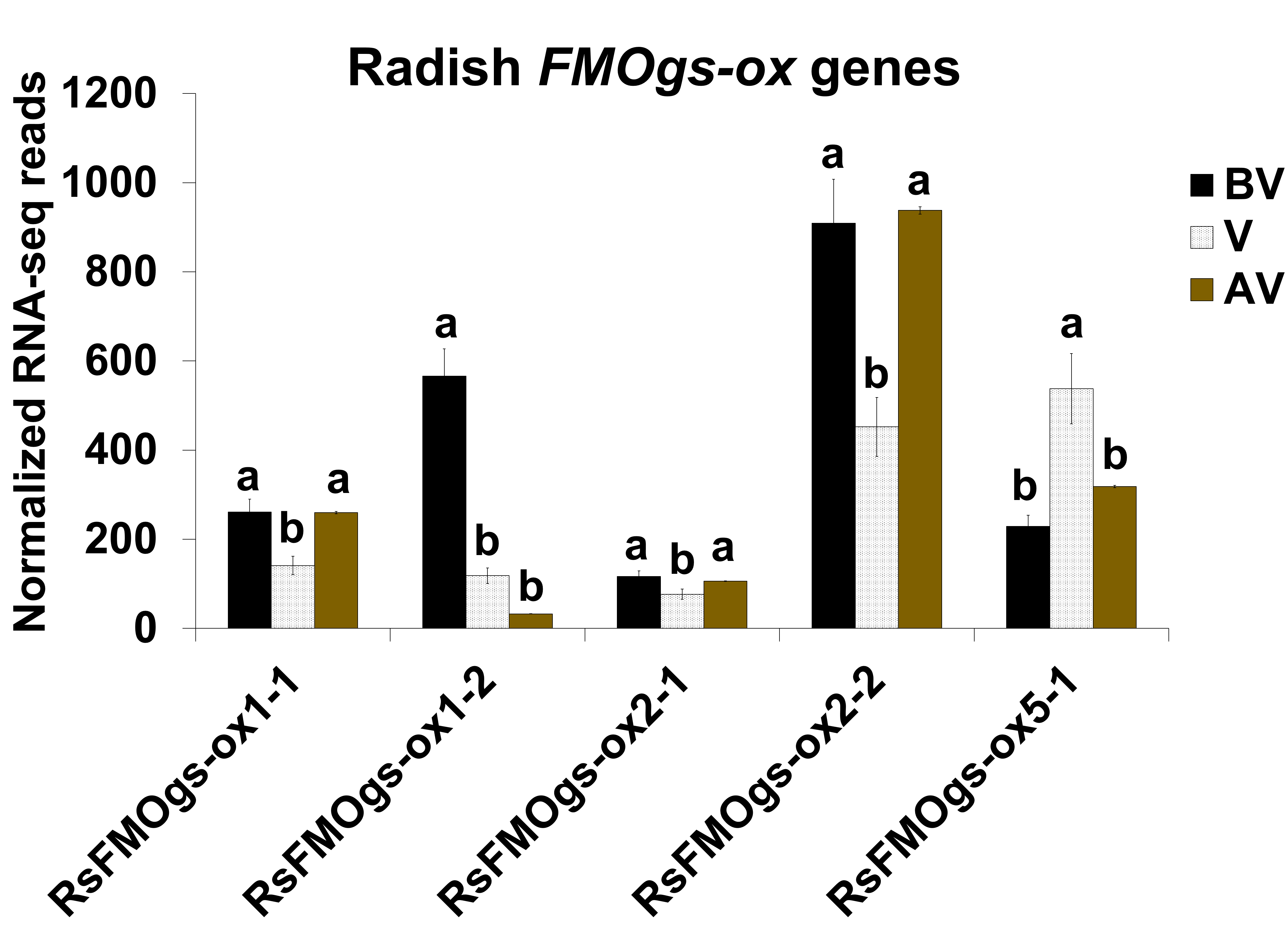
**

**Supplementary Fig. S6** Quantification of RNA-seq reads counts of five *RsFMOgs-oxs* (*RsFMOgs-ox1-1, RsFMOgs-ox1-2, RsFMOgs-ox2-1, RsFMOgs-ox2-2,* and *RsFMOgs-ox5-1*) at three time point (BV: before vernalized, V: vernalized, AV: after vernalized). Data were presented as mean ± standard deviation (SD) (n=3). Statistically significant differences were determined by one-way ANOVA and Tukey’s post hoc test (*p* < 0.05).

**Supplementary Table S1.** Total 93 GSL metabolic pathway related genes in radish.

| **GSLs Groups** | **Biosynthetics Steps** | **Genes** | ***Arabidopsis thaliana (TAIR)*** | ***Raphanus Sativus*** | **NODAI ID** |
| --- | --- | --- | --- | --- | --- |
| **Aliphatic GSLs** | **Transcription factors** | MYB28 | AT5G61420.2 | RsMYB28a | RSG16088.t1 |
|  |  |  |  | RsMYB28b | RSG23384.t1 |
|  |  |  |  | RsMYB28c | RSG53581.t1 |
|  |  | MYB29 | AT5G07690.1 | RsMYB29a | RSG00789.t1 |
|  |  |  |  | RsMYB29b | RSG09585.t1 |
|  |  | MYB76 | AT5G07700.1 |  | - |
|  |  | MYB115 | AT5G40360.1 |  | - |
|  |  | MYB118 | AT3G27785.1 |  | - |
|  | **Side-chain elongation** | BCAT4 | AT3G19710.1 | RsBCAT4 | RSG13682.t1 |
|  |  | BCAT3 | AT3G49680.1 | RsBCAT3a | RSG45676.t1 |
|  |  |  |  | RsBCAT3b | RSG51233.t1 |
|  |  | BAT5 | AT4G12030.2 | RsBAT5 | RSG43366.t1 |
|  |  | MAM1 | AT5G23010.1 | RsMAM1a | RSG11180.t1 |
|  |  |  |  | RsMAM1b | RSG13852.t1 |
|  |  |  |  | RsMAM1c | RSG24637.t1 |
|  |  | MAM3 | AT5G23020 |  | - |
|  |  | IPMI-LSU1 | AT4G13430.1 | RsIPMI-LSU1 | RSG39553.t1 |
|  |  | IPMI-SSU2 | AT2G43100.1 | RsIPMI-SSU2 | RSG05959.t1 |
|  |  | IPMI-SSU3 | AT3G58990 |  | - |
|  |  | IPMDH1 | AT5G14200.1 | RsIPMDH1 | RSG14423.t1 |
|  |  | IPMDH3 | AT1G31180 |  | - |
|  | **Core structure formation** | CYP79F1 | AT1G16410.1 | RsCYP79F1 | RSG07720.t1 |
|  |  | CYP83A1 | AT4G13770.1 | RsCYP83A1 | RSG12342.t1 |
|  |  | GSTF11 | AT3G03190.1 | RsGSTF11 | RSG37909.t1 |
|  |  | GSTU20 | AT1G78370.1 | RsGSTU20 | RSG28592.t1 |
|  |  | GGP1 | AT4G30530.1 | RsGGP1a | RSG01617.t1 |
|  |  |  |  | RsGGP1b | RSG28117.t1 |
|  |  |  |  | RsGGP1c | RSG30252.t1 |
|  |  | SUR1 | AT2G20610.1 | RsSUR1 | RSG38701.t1 |
|  |  | UGT74B1 | AT1G24100.1 | RsUGT74B1 | RSG35660.t1 |
|  |  | UGT74C1 | AT2G31790.1 | RsUGT74C1a | RSG38352.t1 |
|  |  |  |  | RsUGT74C1b | RSG13253.t1 |
|  |  | ST5c/SOT17 | AT1G18590.1 | RsSOT17a | RSG03557.t1 |
|  |  |  |  | RsSOT17b | RSG12634.t1 |
|  |  | ST5b/SOT18 | AT1G74090.1 | RsSOT18a | RSG07388.t1 |
|  |  |  |  | RsSOT18b | RSG12630.t1 |
|  |  |  |  | RsSOT18c | RSG12631.t1 |
|  |  |  |  | RsSOT18d | RSG12632.t1 |
|  |  |  |  | RsSOT18e | RSG12635.t1 |
|  |  |  |  | RsSOT18f | RSG32297.t1 |
|  |  |  |  | RsSOT18g | RSG32298.t1 |
|  |  |  |  | RsSOT18h | RSG49978.t1 |
|  |  |  |  | RsSOT18i | RSG34220.t1 |
|  | **Secondary modification** | GRS1 | AT1G03410.1 | RsGRS1 | RSG02297.t1 |
|  |  | FMOGS-OX1 | AT1G62570.1 | RsFMOGS-OX1-1 | RSG51376.t1 |
|  |  |  |  | RsFMOGS-OX1-2 | RSG57085.t1 |
|  |  | FMOGS-OX2 | AT1G62540.1 | RsFMOGS-OX2-1 | RSG57158.t1 |
|  |  |  |  | RsFMOGS-OX2-2 | RSG15645.t1 |
|  |  | FMOGS-OX3 | AT1G62560.1 |  | - |
|  |  | FMOGS-OX4 | AT1G62570.1 |  | - |
|  |  | FMOGS-OX5 | AT1G65860.1 | RsFMOGS-OX5-1 | RSG14855.t1 |
|  |  |  |  | RsFMOGS-OX5-2 | RSG28761.t1 |
|  |  | AOP1 | AT4G03070.1 | RsAOP1 | RSG08515.t1 |
|  |  | AOP2 | AT4G03060.1 |  | - |
|  |  | AOP3 | AT4G03050.2 |  | - |
|  |  | GSL-OH | AT2G25450.1 | RsGS-OH | RSG00041.t1 |
|  |  | BZO1 | AT1G65880.1 | RsBZO1 | RSG32181.t1 |
|  | **Transcription factors** | MYB34 | AT5G60890.1 | RsMYB34a | RSG13843.t1 |
|  |  |  |  | RsMYB34b | RSG52510.t1 |
|  |  |  |  | RsMYB34c | RSG19787.t1 |
|  |  | MYB51 | AT1G18570.1 | RsMYB51a | RSG34945.t1 |
|  |  |  |  | RsMYB51b | RSG38989.t1 |
| **Indolic GSLs** |  |  |  | RsMYB51c | RSG03560.t1 |
|  |  | MYB122 | AT1G74080.1 | RsMYB122 | RSG07389.t1 |
|  | **Core structure formation** | CYP79A2 | AT5G05260.1 | RsCYP79A2a | RSG02731.t1 |
|  |  |  |  | RsCYP79A2b | RSG08366.t1 |
|  |  |  |  | RsCYP79A2c | RSG38144.t1 |
|  |  | CYP79B2 | AT5G05260.1 | RsCYP79B2a | RSG17908.t1 |
|  |  |  |  | RsCYP79B2b | RSG23431.t1 |
|  |  |  |  | RsCYP79B2c | RSG33798.t1 |
|  |  | CYP79B3 | AT2G22330.1 | RsCYP79B3 | RSG34152.t1 |
|  |  | CYP83B1 | AT4G31500.1 | RsCYP83B1 | RSG48186.t1 |
|  |  | GSTF9 | AT2G30860.1 | RsGSTF9a | RSG18947.t1 |
|  |  |  |  | RsGSTF9b | RSG32253.t1 |
|  |  | GSTF10 | AT2G30870.1 | RsGSTF10 | RSG32254.t1 |
|  |  | ST5a/SOT16 | AT1G74100.1 | RsSOT16a | RSG07387.t1 |
|  |  |  |  | RsSOT16b | RSG28254.t1 |
|  | **Secondary modification** | CYP81F2 | AT5G57220.1 | RsCYP81F2a | RSG11506.t1 |
|  |  |  |  | RsCYP81F2b | RSG29212.t1 |
|  |  |  |  | RsCYP81F2c | RSG29215.t1 |
|  |  |  |  | RsCYP81F2d | RSG40296.t1 |
|  |  | CYP81F3 | AT4G37400.1 | RsCYP81F3a | RSG10178.t1 |
|  |  |  |  | RsCYP81F3b | RSG23271.t1 |
|  |  | CYP81F4 | AT4G37410.1 | RsCYP81F4 | RSG10180.t1 |
|  |  | IGMT1 | AT1G21100.1 | RsIGMT1a | RSG15203.t1 |
|  |  |  |  | RsIGMT1b | RSG26550.t1 |
|  |  |  |  | RsIGMT1c | RSG59991.t1 |
|  |  | IGMT2 | AT1G21120.1 | RsIGMT2 | RSG15204.t1 |
|  |  | IGMT5 |  | RsIGMT5 | RSG06937.t1 |
| **Breakdown pathway** | | TGG1 | AT5G26000.1 | RsTGG1a | RSG23127.t1 |
|  |  |  |  | RsTGG1b | RSG25390.t1 |
|  |  |  |  | RsTGG1c | RSG30299.t1 |
|  |  |  |  | RsTGG1d | RSG33328.t1 |
|  |  |  |  | RsTGG1e | RSG36879.t1 |
|  |  |  |  | RsTGG1f | RSG40666.t1 |
|  |  |  |  | RsTGG1g | RSG48132.t1 |
|  |  |  |  | RsTGG1h | RSG52100.t1 |
|  |  | TGG2 | AT5G25980.3 | RsTGG2a | RSG30297.t1 |
|  |  |  |  | RsTGG2a | RSG30298.t1 |
|  |  |  |  | RsTGG2c | RSG40189.t1 |
|  |  | PEN2 | AT2G44490.1 | RsPEN2 | RSG16059.t1 |
|  |  | PEN3 | AT1G59870.1 | RsPEN3a | RSG17445.t1 |
|  |  |  |  | RsPEN3b | RSG04372.t1 |
|  |  |  |  | RsPEN3c | RSG05840.t1 |

**Supplementary Table S2.** Expression profiles of 93 GSL metabolic genes in non-vernalized and vernalized radish.

| ***Raphanus Sativus*** | **NODAI ID** | **NV_1** | **NV_2** | **NV_3** | **V_1** | **V_2** | **V_3** | **AV_1** | **AV_2** | **AV_3** |
| --- | --- | --- | --- | --- | --- | --- | --- | --- | --- | --- |
| RsMYB28a | RSG16088.t1 | 1718.35 | 1401.34 | 1680.79 | 2743.95 | 2099.20 | 2209.75 | 903.62 | 889.59 | 889.59 |
| RsMYB28b | RSG23384.t1 | 66.41 | 54.16 | 64.96 | 140.98 | 107.85 | 113.53 | 211.51 | 208.23 | 208.23 |
| RsMYB28c | RSG53581.t1 | 1425.60 | 1162.60 | 1394.44 | 2627.11 | 2009.81 | 2115.66 | 423.53 | 416.95 | 416.95 |
| RsMYB29a | RSG00789.t1 | 4404.89 | 3592.24 | 4308.60 | 4586.16 | 3508.54 | 3693.32 | 489.98 | 482.37 | 482.37 |
| RsMYB29b | RSG09585.t1 | 520.05 | 424.11 | 508.69 | 1108.61 | 848.12 | 892.78 | 86.68 | 85.33 | 85.33 |
| RsBCAT4 | RSG13682.t1 | 4310.96 | 3515.64 | 4216.73 | 3848.46 | 2944.18 | 3099.23 | 1125.10 | 1107.64 | 1107.64 |
| RsBCAT3a | RSG45676.t1 | 2066.67 | 1685.40 | 2021.50 | 2168.36 | 1658.86 | 1746.22 | 1987.49 | 1956.64 | 1956.64 |
| RsBCAT3b | RSG51233.t1 | 887.09 | 723.43 | 867.70 | 1097.03 | 839.26 | 883.45 | 665.88 | 655.54 | 655.54 |
| RsBAT5 | RSG43366.t1 | 1008.45 | 822.41 | 986.41 | 419.39 | 320.85 | 337.74 | 110.55 | 108.84 | 108.84 |
| RsMAM1a | RSG11180.t1 | - | - | - | - | - | - | - | - | - |
| RsMAM1b | RSG13852.t1 | 4367.74 | 3561.95 | 4272.27 | 3486.07 | 2666.94 | 2807.39 | 986.43 | 971.12 | 971.12 |
| RsMAM1c | RSG24637.t1 | 1501.44 | 1224.44 | 1468.62 | 1944.96 | 1487.95 | 1566.31 | 862.98 | 849.59 | 849.59 |
| RsIPMI-LSU1 | RSG39553.t1 | 8381.50 | 6835.22 | 8198.30 | 6287.52 | 4810.13 | 5063.45 | 5930.66 | 5838.61 | 5838.61 |
| RsIPMI-SSU2 | RSG05959.t1 | 1775.95 | 1448.31 | 1737.13 | 1385.11 | 1059.65 | 1115.45 | 478.61 | 471.18 | 471.18 |
| RsIPMDH1 | RSG14423.t1 | 5608.46 | 4573.77 | 5485.86 | 3430.17 | 2624.18 | 2762.38 | 800.26 | 787.84 | 787.84 |
| RsCYP79F1 | RSG07720.t1 | 2852.04 | 2325.87 | 2789.69 | 1533.01 | 1172.79 | 1234.56 | 450.59 | 443.60 | 443.60 |
| RsCYP83A1 | RSG12342.t1 | 3851.87 | 3141.25 | 3767.67 | 4064.15 | 3109.19 | 3272.94 | 925.75 | 911.38 | 911.38 |
| RsGSTF11 | RSG37909.t1 | 1465.11 | 1194.82 | 1433.09 | 1678.10 | 1283.79 | 1351.40 | 431.94 | 425.24 | 425.24 |
| RsGSTU20 | RSG28592.t1 | 15144.11 | 12350.22 | ###### | 13530.76 | ##### | ###### | 5851.29 | 5760.47 | 5760.47 |
| RsGGP1a | RSG01617.t1 | 21471.39 | 17510.19 | ###### | 23830.27 | ##### | ###### | 12904.74 | ###### | 12704.45 |
| RsGGP1b | RSG28117.t1 | 4953.68 | 4039.79 | 4845.40 | 3531.28 | 2701.53 | 2843.80 | 4729.81 | 4656.40 | 4656.40 |
| RsGGP1c | RSG30252.t1 | 13179.39 | 10747.96 | ###### | 7162.46 | 5479.48 | 5768.06 | 7641.93 | 7523.32 | 7523.32 |
| RsSUR1 | RSG38701.t1 | 2593.26 | 2114.84 | 2536.58 | 3029.04 | 2317.30 | 2439.34 | 1307.16 | 1286.87 | 1286.87 |
| RsUGT74B1 | RSG35660.t1 | 2095.30 | 1708.74 | 2049.49 | 1722.95 | 1318.11 | 1387.52 | 669.31 | 658.92 | 658.92 |
| RsUGT74C1a | RSG38352.t1 | 447.69 | 365.09 | 437.90 | 1160.52 | 887.83 | 934.59 | 470.76 | 463.46 | 463.46 |
| RsUGT74C1b | RSG13253.t1 | 2826.28 | 2304.87 | 2764.51 | 2282.07 | 1745.85 | 1837.79 | 335.56 | 330.35 | 330.35 |
| RsSOT17a | RSG03557.t1 | 1578.94 | 1287.65 | 1544.43 | 774.01 | 592.14 | 623.32 | 257.66 | 253.66 | 253.66 |
| RsSOT17b | RSG12634.t1 | 37.06 | 30.22 | 36.25 | 47.35 | 36.22 | 38.13 | 12.46 | 12.27 | 12.27 |
| RsSOT18a | RSG07388.t1 | 1504.98 | 1227.33 | 1472.09 | 1087.74 | 832.15 | 875.98 | 417.13 | 410.66 | 410.66 |
| RsSOT18b | RSG12630.t1 | 65.55 | 53.45 | 64.11 | 96.40 | 73.75 | 77.64 | 24.56 | 24.18 | 24.18 |
| RsSOT18c | RSG12631.t1 | 610.09 | 497.54 | 596.76 | 265.90 | 203.42 | 214.13 | 22.20 | 21.86 | 21.86 |
| RsSOT18d | RSG12632.t1 | 56.19 | 45.82 | 54.96 | 31.72 | 24.26 | 25.54 | 5.72 | 5.64 | 5.64 |
| RsSOT18e | RSG12635.t1 | - | - | - | - | - | - | - | - | - |
| RsSOT18f | RSG32297.t1 | 2987.49 | 2436.34 | 2922.19 | 1117.90 | 855.22 | 900.26 | 712.73 | 701.67 | 701.67 |
| RsSOT18g | RSG32298.t1 | - | - | - | - | - | - | - | - | - |
| RsSOT18h | RSG49978.t1 | - | - | - | - | - | - | - | - | - |
| RsSOT18i | RSG34220.t1 | 22.47 | 18.32 | 21.98 | 3.23 | 2.47 | 2.60 | 0.00 | 0.00 | 0.00 |
| RsGRS1 | RSG02297.t1 | 8973.99 | 7318.40 | 8777.83 | 4296.73 | 3287.12 | 3460.23 | 1464.76 | 1442.03 | 1442.03 |
| RsFMOGS-OX1-1 | RSG51376.t1 | 607.83 | 495.69 | 594.54 | 137.91 | 105.50 | 111.06 | 32.94 | 32.43 | 32.43 |
| RsFMOGS-OX1-2 | RSG57085.t1 | 280.70 | 228.92 | 274.56 | 164.64 | 125.95 | 132.59 | 262.50 | 258.42 | 258.42 |
| RsFMOGS-OX2-1 | RSG57158.t1 | 124.88 | 101.84 | 122.15 | 89.51 | 68.48 | 72.09 | 107.08 | 105.42 | 105.42 |
| RsFMOGS-OX2-2 | RSG15645.t1 | 976.26 | 796.15 | 954.92 | 527.64 | 403.66 | 424.92 | 947.55 | 932.84 | 932.84 |
| RsFMOGS-OX5-1 | RSG14855.t1 | 246.06 | 200.67 | 240.68 | 627.43 | 480.00 | 505.28 | 321.57 | 316.58 | 316.58 |
| RsFMOGS-OX5-2 | RSG28761.t1 | - | - | - | - | - | - | - | - | - |
| RsAOP1 | RSG08515.t1 | 82.37 | 67.17 | 80.57 | 60.28 | 46.11 | 48.54 | 168.20 | 165.59 | 165.59 |
| RsGS-OH | RSG00041.t1 | 29.79 | 24.30 | 29.14 | 92.44 | 70.72 | 74.44 | 17.51 | 17.24 | 17.24 |
| RsBZO1 | RSG32181.t1 | - | - | - | - | - | - | - | - | - |
| RsMYB34a | RSG13843.t1 | 110.68 | 90.26 | 108.26 | 140.01 | 107.11 | 112.75 | 178.56 | 175.79 | 175.79 |
| RsMYB34b | RSG52510.t1 | 10.83 | 8.83 | 10.59 | 31.60 | 24.17 | 25.44 | 5.39 | 5.30 | 5.30 |
| RsMYB34c | RSG19787.t1 | 23.84 | 19.44 | 23.32 | 202.54 | 154.95 | 163.11 | 1.35 | 1.33 | 1.33 |
| RsMYB51a | RSG34945.t1 | 131.37 | 107.14 | 128.50 | 119.82 | 91.66 | 96.49 | 336.00 | 330.78 | 330.78 |
| RsMYB51b | RSG38989.t1 | 132.86 | 108.35 | 129.95 | 139.45 | 106.69 | 112.30 | 515.75 | 507.74 | 507.74 |
| RsMYB51c | RSG03560.t1 | 11.90 | 9.70 | 11.64 | 15.21 | 11.63 | 12.25 | 19.90 | 19.59 | 19.59 |
| RsMYB122 | RSG07389.t1 | 61.11 | 49.83 | 59.77 | 161.02 | 123.19 | 129.67 | 154.53 | 152.13 | 152.13 |
| RsCYP79A2a | RSG02731.t1 | - | - | - | - | - | - | - | - | - |
| RsCYP79B2a | RSG17908.t1 | 192.95 | 157.35 | 188.73 | 272.67 | 208.60 | 219.58 | 278.20 | 273.89 | 273.89 |
| RsCYP79B2b | RSG23431.t1 | 33.28 | 27.14 | 32.55 | 44.48 | 34.03 | 35.82 | 16.16 | 15.91 | 15.91 |
| RsCYP79B2c | RSG33798.t1 | 8.30 | 6.77 | 8.12 | 30.43 | 23.28 | 24.51 | 20.23 | 19.91 | 19.91 |
| RsCYP79B3 | RSG34152.t1 | 57.85 | 47.18 | 56.59 | 45.71 | 34.97 | 36.81 | 54.22 | 53.38 | 53.38 |
| RsCYP83B1 | RSG48186.t1 | 5571.82 | 4543.89 | 5450.03 | 6523.69 | 4990.81 | 5253.64 | 7738.38 | 7618.27 | 7618.27 |
| RsGSTF9a | RSG18947.t1 | 17635.16 | 14381.70 | ###### | 16005.32 | ##### | ###### | 6141.89 | 6046.56 | 6046.56 |
| RsGSTF9b | RSG32253.t1 | 8543.83 | 6967.60 | 8357.07 | 7228.14 | 5529.73 | 5820.95 | 4710.89 | 4637.77 | 4637.77 |
| RsGSTF10 | RSG32254.t1 | 16705.02 | 13623.15 | ###### | 12504.25 | 9566.10 | ###### | 14590.97 | ###### | 14364.50 |
| RsSOT16a | RSG07387.t1 | 733.06 | 597.82 | 717.03 | 1246.24 | 953.41 | 1003.62 | 778.49 | 766.41 | 766.41 |
| RsSOT16b | RSG28254.t1 | 804.46 | 656.05 | 786.87 | 1236.57 | 946.01 | 995.83 | 1147.97 | 1130.15 | 1130.15 |
| RsCYP81F2a | RSG11506.t1 | 11.86 | 9.67 | 11.60 | 30.90 | 23.64 | 24.89 | 42.14 | 41.48 | 41.48 |
| RsCYP81F2b | RSG29212.t1 | 20.91 | 17.05 | 20.45 | 4.04 | 3.09 | 3.25 | 1.35 | 1.33 | 1.33 |
| RsCYP81F2c | RSG29215.t1 | 14.04 | 11.45 | 13.73 | 5.04 | 3.86 | 4.06 | 22.54 | 22.19 | 22.19 |
| RsCYP81F2d | RSG40296.t1 | 467.47 | 381.23 | 457.25 | 247.57 | 189.39 | 199.37 | 144.41 | 142.17 | 142.17 |
| RsCYP81F3a | RSG10178.t1 | 40.44 | 32.98 | 39.56 | 38.15 | 29.18 | 30.72 | 13.17 | 12.96 | 12.96 |
| RsCYP81F3b | RSG23271.t1 | 50.08 | 40.84 | 48.98 | 5.45 | 4.17 | 4.39 | 18.85 | 18.56 | 18.56 |
| RsCYP81F4 | RSG10180.t1 | 14.59 | 11.90 | 14.27 | 349.38 | 267.28 | 281.36 | 164.09 | 161.54 | 161.54 |
| RsIGMT1a | RSG15203.t1 | 73.30 | 59.78 | 71.70 | 317.39 | 242.81 | 255.60 | 280.50 | 276.15 | 276.15 |
| RsIGMT1b | RSG26550.t1 | 55.29 | 45.09 | 54.08 | 69.28 | 53.00 | 55.79 | 60.30 | 59.36 | 59.36 |
| RsIGMT1c | RSG59991.t1 | 238.26 | 194.30 | 233.05 | 351.02 | 268.54 | 282.68 | 300.57 | 295.90 | 295.90 |
| RsIGMT2 | RSG15204.t1 | 112.19 | 91.49 | 109.74 | 353.71 | 270.60 | 284.85 | 158.44 | 155.98 | 155.98 |
| RsIGMT5 | RSG06937.t1 | 2430.02 | 1981.71 | 2376.90 | 3393.37 | 2596.02 | 2732.74 | 3181.93 | 3132.54 | 3132.54 |
| RsTGG1a | RSG23127.t1 | 15462.83 | 12610.14 | ###### | 6351.22 | 4858.86 | 5114.75 | 2118.82 | 2085.93 | 2085.93 |
| RsTGG1b | RSG25390.t1 | 572.93 | 467.23 | 560.40 | 703.58 | 538.26 | 566.60 | 45.19 | 44.49 | 44.49 |
| RsTGG1c | RSG30299.t1 | 31402.70 | 25609.31 | ###### | 2242.17 | 1715.32 | 1805.66 | 2611.20 | 2570.67 | 2570.67 |
| RsTGG1d | RSG33328.t1 | 19167.99 | 15631.74 | ###### | 3712.50 | 2840.17 | 2989.75 | 5047.71 | 4969.37 | 4969.37 |
| RsTGG1e | RSG36879.t1 | 2110.46 | 1721.11 | 2064.33 | 5281.99 | 4040.87 | 4253.68 | 719.57 | 708.40 | 708.40 |
| RsTGG1f | RSG40666.t1 | 182.66 | 148.97 | 178.67 | 815.79 | 624.10 | 656.97 | 36.06 | 35.50 | 35.50 |
| RsTGG1g | RSG48132.t1 | 1374.26 | 1120.72 | 1344.22 | 504.65 | 386.07 | 406.40 | 195.65 | 192.61 | 192.61 |
| RsTGG1h | RSG52100.t1 | 1651.44 | 1346.77 | 1615.34 | 818.33 | 626.05 | 659.02 | 1052.55 | 1036.21 | 1036.21 |
| RsTGG2a | RSG30297.t1 | 7782.80 | 6346.97 | 7612.68 | 819.98 | 627.31 | 660.35 | 853.91 | 840.66 | 840.66 |
| RsTGG2a | RSG30298.t1 | 5755.45 | 4693.64 | 5629.64 | 505.96 | 387.07 | 407.46 | 466.32 | 459.08 | 459.08 |
| RsTGG2c | RSG40189.t1 | 1098.81 | 896.09 | 1074.79 | 87.32 | 66.80 | 70.32 | 26.58 | 26.17 | 26.17 |
| RsPEN2 | RSG16059.t1 | 4146.14 | 3381.23 | 4055.51 | 6227.05 | 4763.87 | 5014.76 | 10892.60 | ###### | 10723.53 |
| RsPEN3a | RSG17445.t1 | 2373.64 | 1935.74 | 2321.76 | 9555.34 | 7310.11 | 7695.09 | 15169.41 | ###### | 14933.97 |
| RsPEN3b | RSG04372.t1 | 2099.20 | 1711.92 | 2053.31 | 2480.83 | 1897.90 | 1997.86 | 10045.21 | 9889.29 | 9889.29 |
| RsPEN3c | RSG05840.t1 | 2968.28 | 2420.67 | 2903.40 | 5857.87 | 4481.43 | 4717.44 | 11425.63 | ###### | 11248.29 |

**Supplementary Table S3.** Total reads and mapped reads on the radish reference genome.

| **Sample ID** | **Total read bases (bp)** | **Total reads** | **Overall mapping rate (% of total reads)** | **GC(%)** | **AT(%)** | **Q20(%)** | **Q30(%)** |
| --- | --- | --- | --- | --- | --- | --- | --- |
| BV-1 | 6,307,102,285 | 62,494,762 | 84.70% | 47.029 | 52.97 | 98.52 | 95.253 |
| BV-2 | 5,139,338,352 | 50,917,048 | 81.00% | 46.551 | 53.45 | 98.535 | 95.268 |
| BV-3 | 6,134,760,276 | 60,774,622 | 81.40% | 47.54 | 52.46 | 98.597 | 95.475 |
| V-1 | 6,786,091,552 | 67,238,578 | 80.90% | 47.241 | 52.76 | 98.594 | 95.465 |
| V-2 | 5,306,160,447 | 52,561,526 | 81.10% | 47.17 | 52.83 | 98.669 | 95.58 |
| V-3 | 5,358,869,348 | 53,091,696 | 80.90% | 47.116 | 52.88 | 98.56 | 95.34 |
| AV-1 | 6,199,318,714 | 61,431,526 | 80.60% | 47.208 | 52.79 | 98.669 | 95.637 |
| AV-2 | 6,422,678,988 | 63,696,338 | 78.70% | 46.587 | 53.41 | 97.923 | 93.912 |
| AV-3 | 4,933,480,084 | 48,892,412 | 82.50% | 48.068 | 51.93 | 98.793 | 95.998 |

**Supplementary Table S4.** List of primers used in this study

| **Primer name** | **Sequence** | **Usage** |
| --- | --- | --- |
| RsACTIN_F | ATCAGGAAGGACTTGTACGGTAAC | RT-qPCR |
| RsACTIN_R | GCTGAGGGAAGCAAGAATGGAACC | RT-qPCR |
| RsGRS1_F | GTTCCTCCTGTCCCTGGATG | RT-qPCR |
| RsGRS1_R | ATGCGCTAGCACCCTATGCT | RT-qPCR |
| RsGRS1_promoter_F | GAAAAACATATTTGAAGGGTATTTGATTTATTTATTGTAGAAGAG | ChIP-qPCR |
| RsGRS1_promoter_R | GTAAGGTTATTTCGAGGTGGCACAAC | ChIP-qPCR |
| RsGRS1_TSS_F | GAGAAACTGACACTAAGGTAAATCATACTTTTATGC | ChIP-qPCR |
| RsGRS1_TSS_R | TTAAACGAGTAATAAACGACTTTTCGTCTCAGT | ChIP-qPCR |
| RsGRS1_Gene body_F | GCGTCAAGTCTCAGATGATGTTGGGC | ChIP-qPCR |
| RsGRS1_Gene body_R | CCAGGGACAGGAGGAACATCAAACC | ChIP-qPCR |
| RsACTIN_F | CAACTCTCCGGCTATGTATGTCGCTATCC | ChIP-qPCR |
| RsACTIN_R | CGTAGATCGGCACAGTGTGAGACAC | ChIP-qPCR |

**Supplementary Table S5.** List of standard compounds of aliphatic GSL used in this study

| **Compounds Name** | **Molecular Formula** | **Groups** | **Cat. No** |
| --- | --- | --- | --- |
| Progoitrin (PGT) | C_11_H_18_KNO_10_S_2_ | Aliphatic | 21087-77-4* |
| Glucoraphanin (GRA) | C_12_H_22_KNO_10_S_3_ | Aliphatic | 21414-41-5 |
| Glucoraphenin (GRE) | C_12_H_20_KNO_10_S_3_ | Aliphatic | 108844-81-1 |
| Sinigrin (SIN) | C_10_H_16_KNO9S_2_ | Aliphatic | 3952-98-5 |
| Glucoalyssin (GAS) | C_13_H_24_KNO_10_S_3_ | Aliphatic | 499-37-6 |
| Gluconapin (GNP) | C_11_H_18_KNO_9_S_2_ | Aliphatic | 245550-57-6 |
| Glucobrassicanapin (GBN) | C_12_H_20_KNO_9_S_2_ | Aliphatic | 245550-58-7 |
| Glucoerucin (GER) | C_12_H_22_KNO_9_S_3_ | Aliphatic | 15592-37-7 |
| Glucoraphasatin (GRH) | C_12_H_20_KNO_9_S_3_ | Aliphatic | 28463-23-2 |
| Glucobrassicin (GBS) | C_16_H_19_KN_2_O_9_S_2_ | Indolic | 4356-52-9 |
| 4-Methoxyglucobrassicin (4-MTGB) | C_17_H_21_KN_2_O_10_S_2_ | Indolic | 833327-21-3 |
| 4-Methoxyglucobrassicin (4-HGB) | C_16_H_19_KN_2_O_10_S_2_ | Indolic | 833327-20-2 |
| Neoglucobrassicin (NGB) | C_17_H_21_KN_2_O_10_S_2_ | Indolic | 5187-84-8 |
| Gluconasturtiin (GNT) | C_15_H_20_KNO_9_S_2_ | Aromatic | 18425-76-8 |

*All the GSLs standard compounds was purchased from Phytoplan (Germany), except the sinigrin that was purchased from Sigma-aldrich (USA).
